# Generative AI in drug repurposing and biomarker discovery: a multimodal approach

**DOI:** 10.3389/fbinf.2026.1755412

**Published:** 2026-03-20

**Authors:** K. Saranya, Emerson Raja Joseph, Ts. Kalaiarasi, M. Karthiga

**Affiliations:** 1 Department of Computer Science & Engineering, Bannari Amman Institute of Technology, Erode, Tamilnadu, India; 2 Centre for Advanced Analytics, Multimedia University, Melaka, Malaysia; 3 Faculty of Engineering and Technology Centre for Advanced Analytics, OE of Artificial Intelligence, Multimedia University, Melaka, Malaysia; 4 Faculty of Information Science and Technology, Multimedia University, Melaka, Malaysia; 5 Department of Computer Science and Engineering, Bannari Amman Institute of Technology, Sathyamangalam, Tamilnadu, India

**Keywords:** biomarker discovery, drug repurposing, generative artificial intelligence, heterogeneous graph neural networks, model-agnostic meta-learning, multi-omics integration

## Abstract

**Introduction:**

Computational drug repurposing has been widely explored using similarity-based methods, network diffusion, matrix factorization, deep learning, and graph neural networks (GNNs). However, recent heterogeneous GNN models, such as TxGNN and GAT-based models, demonstrate serious limitations for real-world biomedical applications, including poor generalization to sparsely annotated diseases, limited disease-level adaptation, and inability to effectively combine heterogeneous evidence from curated databases, multi-omics profiles, and unstructured biomedical literature.

**Methods:**

This article proposes a heterogeneous attention-based meta-learning graph neural network named HAMGNN, which employs three major innovations: (i) relation-sensitive multi-head attention to prioritize biologically significant interactions among heterogeneous edge types, (ii) a disease-focused meta-learning framework enabling rapid adaptation to newly observed or under-informed diseases, and (iii) a literature-enhanced knowledge graph construction pipeline encoding high-confidence, LLM-extracted therapeutic information. The model was tested on a large multimodal biomedical knowledge graph assembled from DrugBank, DisGeNET, and Hetionet, comprising more than 2.2 million edges, using a stringent disjoint disease-based (cold-start) evaluation protocol.

**Results:**

HAMGNN achieved a receiver operating characteristic–area under the curve (ROC–AUC) of 0.98 and precision of 0.95, representing a 10%–15% improvement over TxGNN and GAT-GNN on unseen disease generalization. Translational applicability was demonstrated through Alzheimer’s disease and Long COVID case studies, identifying clinically plausible repurposing candidates and disease-associated biomarker signatures via mechanistic pathways.

**Discussion:**

HAMGNN offers a generalized, biologically grounded, and unified framework for evidence-based drug repurposing and biomarker discovery in complex and emerging diseases.

## 1 Introduction

The current state of biomedical research is characterized by large datasets that describe drug properties, disease relationships, gene expression, biological pathways, and multi-omics signatures (Vora et al., 2025). Resources like DrugBank, DisGeNET, Hetionet, LINCS L1000, TCGA, ADNI, and massive biomedical knowledge graphs provide information that describes millions of molecular interactions in the form of both structured and unstructured data. All these datasets contain drug–target interactions, a network of gene regulation, disease ontologies, protein functions, side-effect data, transcriptomic perturbations, imaging biomarkers, and clinical outcome data (Zeng et al., 2022a). The associated graph topology may often consist of non-homogeneous node types, e.g., drugs, genes, diseases, pathways, and phenotypes, that are related by multi-relational edges based on curated databases, high-throughput screens, and scientific literature. These massive multimodal datasets allow comprehensive modeling of disease processes, therapeutic responses, and molecular signatures, which are the basis of computational drug repurposing and biomarker discovery.

Biomedical datasets are heterogeneous, incomplete, and noisy due to their richness. The data are obtained through a variety of sources involving diverse experimental platforms, annotation standards, and quality-control protocols, which cause discrepancies and missing connections (Ogidi et al., 2025). Associations between drugs and diseases, between genes, and between pathways are frequently sparse, particularly with infrequent diseases or recently emerged diseases such as Long COVID. Additional complexity of high-dimensional omics is characterized by batch effects, redundant measurements, and variable sample quality. The data derived from the literature are ambiguous due to variations in terminologies, use of synonyms, and, to a certain extent, variation in context. Furthermore, the amount of relational information among molecular entities is often skewed: hundreds of interactions are frequently annotated with a drug, whereas only a handful of annotated interactions exist with a drug (Gangwal et al., 2024). These issues decrease the reliability of models and increase the difficulty of integrating features and generalizing therapeutic predictions across diseases.

Traditional methods of computational drug repositioning and biomarker discovery are based on manually engineered features in conjunction with classical machine learning models. Some of the common strategies encompass similarity-based drug ranking, network propagation algorithms, matrix factorization, and statistical enrichment analyses (Mariam et al., 2024). The common features extracted are chemical descriptors, target profiles, disease–gene association, or pathway membership, which are then fed to classifiers like support vector machines, random forests, logistic regression, or simple neural networks. These approaches consider most of the input data to be independent vectors and do not model higher-order biomedical relationships. Moreover, classical models involve preset similarity measures or manually engineered features, and thus, they are strongly performance sensitive to domain knowledge (Romanelli et al., 2024). These methods are limited to small-scale problems, and they fail to deal with complex graph structure, multimodal interactions, and dynamic biological behavior.

Conventional feature-based methods have major limitations when used on large heterogeneous biomedical graphs. Features engineered manually do not reflect the contextual associations of drugs, genes, and diseases, leading to the loss of the interaction-level biological content. Similarity measures are unable to capture nonlinear relationships or multistage biological relationships (Yingngam, 2024). Classical classifiers do not have the ability to combine multi-relational data, giving them weak predictive ability in situations that require reasoning about complicated graph representations. Most available models also presuppose the same data distributions, which is why they are not applicable to sparse, imbalanced datasets. The ability to generalize to novel diseases, novel pathogens, or poorly studied phenotypes is still unsatisfactory, as the conventional techniques do not reliably apply learned information to different disease settings. These deficiencies underscore the importance of highly scalable architectures that can capture structural, relational, and multimodal patterns (Lilhore et al., 2025).

Although the computation methods applied to drug repurposing are rather diverse, with transcriptomic signature matching, inference by similarity, and inferring the patterns of the tensors being processed, graph neural networks have become the best way to model complicated biological interactions (Vincenzo, 2024). However, current GNN models are frequently based on dense relational connectivity, which is not applicable to a significant percentage of rare or emergent diseases. For example, TxGNN is highly sensitive to network proximity assumptions and may not be effective in characterizing new conditions or pathogens, and GAT-based models do not generally interact with heterogeneous multi-omics and literature-based evidence effectively (Zhang et al., 2025). The HAMGNN framework mitigates these drawbacks by integrating heterogeneous relation-filtering attention with meta-learning-based disease adaptation. This approach enhances generalization across sparse and heterogeneous therapeutic landscapes.

The suggested study is carried out in a hierarchical pipeline that will help to combine different data types and prediction operations. The initial step builds a heterogeneous biomedical graph using drug, gene, disease, pathway, and multi-omics data so that the entities have comparable feature representations. The second phase uses the HAMGNN architecture with a multi-head attention and meta-learning to forecast drug–disease relationships and compare the results with the existing TxGNN and GAT-GNN models. The third phase presents a conditional generative module in generating new drug combinations that are maximally synergistic in therapeutic effect. The fourth step employs domain-optimized generative large language models (LLMs) to identify new drug–disease knowledge from textual evidence in scientific literature. This methodological use of AI is strictly for knowledge graph augmentation and was not involved in the drafting of this article. The fifth step combines multi-omics data to determine predictive biomarkers in relation to disease development or response to treatment. Validation is done through pathway enrichment, molecular docking, and correlation of clinical output, and then an ethical assessment is done covering transparency, privacy protection, and fair accessibility. This landmap guarantees a logical series between the dataset construction and translational evaluation.

The main contributions of the proposed work:

- A heterogeneous, attention-based meta-learning GNN (HAMGNN) designed for improved drug–disease association prediction.
- Integration of multi-relational biomedical data into a unified graph framework for enhanced contextual representation.
- A conditional generative module for discovering synergistic drug combinations with higher predictive accuracy.
- Literature-driven extraction of hidden therapeutic relationships using domain-optimized large language models.
- Multi-omics fusion for robust biomarker identification across complex diseases.
- A complete *in silico* validation pipeline featuring pathway analysis, molecular docking, and clinical outcome correlation.

Part II describes the current graph-based and generative AI methods of drug repurposing and biomarker discovery and outlines their benefits in terms of representing features and combinations of knowledge, with drawbacks due to sparsity, low generalization capacity, and inability to think multimodally. Section 3 outlines the proposed architecture with the description of the workflow of the heterogeneous attention-based meta-learning GNN, the generative module of heterogeneous drug-combination generation, the literature-mining component, and the multi-omics integration pipeline of biomarker prediction. Section 4 presents the results of the experiment, which included better forecasting drug–disease relationships, drug-combination quality, literature-extracted therapeutic correlations, and performance of biomarkers, as well as comparative performance to models like TxGNN and GAT-GNN. Section 5 concludes the study by presenting the contributions to computational drug repurposing and biomarker discovery and outlining the future directions of promoting multimodal learning, scalability, and clinical translation.

## 2 Literature survey

Computational drug-repurposing methods can be broadly grouped into three methodological paradigms, each with distinct strengths and limitations that motivate the design of HAMGNN.

- Traditional and network-based drug repurposing:

The first computational drug-repurposing methods were mostly based on similarity-based ranking, random walks, and matrix factorization (e.g., Node2vec) on biological networks. Although they can work well in a comparatively dense graph, the basic assumptions in these kinds of approaches imply homogeneous relationships and compress very different biomedical interactions into the same type of edge. Consequently, they do not maintain the unique semantics of drug–target, gene–disease, and pathway-level interactions, and their inductive quality is restricted by the transductive nature of inferred disease concepts in unseen diseases or in sparsely annotated conditions.

Himmelstein et al. (2017) proposed Hetionet, a medical scale system of knowledge integration that incorporated drugs, diseases, genes, and pathways into a heterogeneous network. The network features were manually engineered to infer drug–disease associations where logistic regression is used, which allows interpretation and biological transparency. Nonetheless, this method is based on fixed feature construction and linear models, thereby limiting its ability to learn nonlinear higher-order dependencies and scale to changing biomedical graphs.

Zhou et al. (2014) created a human symptom disease network on the premise of clinical as well as phenotypic similarity, which demonstrated latent disease-disease connections and comorbidity patterns. Although this framework can be useful in the classification of diseases, it is indirectly related to therapeutic inference because it does not directly model drug interactions or molecular processes, and thus limits the use of the framework to drug repurposing tasks.

A network diffusion-based model of drug repurposing was proposed by Cheng et al. (2019), which spreads the signal in drug–target–disease networks and verifies the predictions on the population-scale real-world data. Although such a diffusion paradigm is better in enhancing translational relevance than strictly *in silico* methods, it is based on handcrafted propagation rules and assumes a static network, allowing limited flexibility to compute multi-relational semantics or adaptation to new diseases with only a small number of annotations.

Together, these classical network-based methods show that similarity measures, diffusion methods, and matrix factorization methods can be used to capture coarse relational patterns. However, they are not yet sufficient to do modern drug repurposing because of their failure to jointly model heterogeneous biomedical relations, their inability to generalize inductively and in a few-shot fashion, and their need to rely on manually engineered features. These shortcomings drive the shift to relation-aware, learning-based graph models that can engage in adaptive reasoning in sparse, multimodal biomedical environments.

ii. Graph neural networks and knowledge graph learning:

Graph neural networks (GNNs) presented message-passing operations, which enhanced representation learning on graph-structured biomedical data to a large extent. Nevertheless, initial homogeneous GNNs, such as graph convolutional networks (GCNs) and graph attention networks (GATs), implicitly assume that there is one type of edge, and information on all neighbors is aggregated without maintaining the meaning of different biomedical relations. Such homogenization in drug–disease knowledge graphs homogenizes drug–target, gene–disease, and pathway interactions in a single stream of message, introducing biological noise and decreasing interpretability. Despite performing competitively on typical link-prediction datasets, these models are based on dense neighborhood connectivity, and they thus run sharply in cold-start conditions, where diseases lack known drug interactions or have only a few.

They have been replaced with graph convolutional networks (GCNs) suggested by Kipf and Welling (2017) to execute localized spectral convolutions by summing up normalized neighbor features in a transductive, semi-supervised context. Although computationally efficient, GCNs use the same transformation functions across all the edges, which is equivalent to the homogeneity of the relations. GATs were proposed by Veličković et al. (2018) to substitute homogeneous aggregation with attention coefficients that are learned throughout the neighbors. Despite the improvements in attention-based neighbor weighting and resilience to noisy connections, GATs continue to be used with homogeneous message passing and have no way of differentiating between biologically different types of relations.

To overcome the multi-relational structure, relational GNNs were created. Zitnik et al. (2018) set out a proposal, Decagon, a type of relational graph convolutional framework that characterizes the polypharmacy side effects based on the edge-type-specialized parameter on a multi-relational drug–protein graph. In the same way, Schlichtkrull et al. (2018) proposed a modification of GCNs, relational graph convolutional networks (R-GCNs), that add relation-specific transformation matrices to the message flow. These models maintain edge semantics better than homogeneous GNNs but require that the number of observations per type of relation is sufficiently large. They can frequently have problems with parameter explosion and over-smoothing in large, sparse biomedical graphs.

Inductive GNNs like GraphSAGE, introduced by Hamilton et al. (2017), go a step further by allowing message passing by sampling and aggregating features from the neighborhood and by predicting previously unseen nodes. Although this inductive power is beneficial to graph evolutions, the GraphSAGE itself is not specific to heterogeneous biomedical applications because it treats relations equally without any explicit manipulation.

More recent heterogeneous GNN models that are disease-centric make attempts to address these problems by explicitly modeling diseases as focal prediction tasks and organizing message passing around disease-centric neighborhoods. Although they perform better in the few-shot case, current disease-centric models usually use fixed relation weighting schemes/curated graph structures, which restricts their ability to adapt to sparse, noisy, or dynamically augmented biomedical knowledge graphs.

Altogether, current GNN paradigms demonstrate a natural evolution of homogeneous aggregation to relation-conscious models, but they are similarly limited by simplistic message-passing models, weakness in sparse regimes, and lack of adaptation to the disease level. These constraints drive architectures that concurrently combine relation-aware attention, inductive generalization, and disease-specific adaptation, which are the design objectives of the proposed HAMGNN framework.

iii. Heterogeneous biomedical knowledge graphs:

Recent frameworks include TxGNN, which applies disease-centric learning on heterogeneous graphs, achieving state-of-the-art performance. However, they are limited to structured databases and fail to use unstructured scientific literature and multimodal omics evidence, limiting flexibility and biological scale.

Zeng et al. (2022b) suggested a framework of learning based on knowledge graphs to repurpose tested drugs against COVID-19 by combining open biomedical data resources with a single heterogeneous graph. Their approach is the addition of deep learning to the knowledge graph by predicting probable therapeutic leads based on drug–target–disease relationships, which is useful in responding immediately to a societal health disaster. One of the strong points of this work is its ability to use publicly available, large-scale data to create timely and biologically reasonable predictions.

Fu et al. (2022) proposed a disease-centric heterogeneous graph neural network named TxGNN for use in few-shot drug discovery. Its methodology is a clear model of diseases in the form of tasks, and it uses meta-learning to facilitate fast adaptation in situations where a small number of drug–disease relationships are known. This design solves a severe shortcoming of traditional GNNs when they are applied to rare diseases and offers high performance in cold-start settings.

Yu et al. (2023) designed a heterogeneous graph learning platform in drug discovery that incorporates a variety of biomedical entities and relation types in one graph representation. Their method is based on using graph neural networks to learn multifaceted patterns of interaction between drugs, targets, and diseases to enhance predictive performance compared to non-heterogeneous models.

Zhao et al. (2024) suggest a disease-aware graph learning method that improves drug repurposing by introducing disease-related representations in the graph neural networks. The model enhances the relevance of predicted associations of drugs and diseases by conditioning message passing on disease context. This model has better disease specificity than generic GNN models.

iv. Cold-start and few-shot learning in drug discovery:

Meta-learning has become a conceptually sound approach to resolving the cold-start problem in rare and emerging diseases, in which the labeled drug–disease associations are either sparse or non-existent. Finn et al. (2017) proposed a framework model-agnostic meta-learning (MAML), an optimization-based system that solves training as a bi-level optimization problem, which allows a model to quickly adapt to new tasks with only a few gradient updates. Instead of maximizing training on a fixed dataset, MAML learns an initialization that is directly optimized to rapidly adapt to individual tasks, which results in it being especially applicable to the case of data sparsity.

Based on this paradigm, Wang et al. (2023) trained meta-learning on few-shot drug discovery, with each prediction task, including drug–target or drug–disease association prediction, being an independent task. With each discovery problem as a separate learning episode, their solution enables knowledge to be transferred to similar biomedical tasks and remain adaptable to new low-data problems. This task formulation makes it more robust in environments where standard models of supervised learning overfit to objects under study.

This notion was further developed by Huang et al., who modeled the different diseases as distinct tasks during the discovery of rare disease drugs (Huang et al., 2022). Their structure integrates shared representation learning with disease-specific adaptation and allows generalizing disease-specific mechanisms across heterogeneous mechanisms, but it is also adaptable to limited disease-specific evidence. Such a disease-as-task formulation is very similar to drug repurposing in the real world, where forecasting is needed when little or no therapeutic annotation of the disease is available.

All these studies point to the deficiency of the traditional supervised GNNs under disjoint disease assessment protocols. In GNN training, standard GNN learning assumes that every disease seen in inference was seen in training, which is frequently implicitly optimized by common neighborhood structure or exposure to node identities. In cases where diseases are not repurposed at all, as in realistic repurposing of rare or emerging conditions, the supervised models have no mechanism for fast adaptation and severely degrade their performance. Meta-learning can overcome this shortcoming by directly training models to learn across disease-specific tasks, in which case cold-start drug repurposing can be guided by principle and encourage its inclusion in the proposed HAMGNN framework.

v. Multimodal and multi-omics integration:

Multimodal and multi-omics integration has been developed at various levels of abstraction, and those levels have significant implications on drug–disease modeling. Concatenation of heterogeneous data modalities at the feature level, that is, feature-level fusion, has been used extensively in initial clinical prediction research. An example is the study by Huang et al. (2020), which juxtaposes medical imaging with electronic health records via modality-specific encoders with subsequent feature aggregation and refined prediction on clinical assignments. Although useful in supervised prediction of outcomes, these methods assume modalities are discrete groups of features and do not model interactions between entities biologically, which restricts their use to mechanistic drug repurposing.

More sophisticated methods embrace representation-level fusion, where each modality encoder learns latent representations that are then merged or matched. This strategy was used by Mathema et al. (2023) to answer the question of precision cancer medicine, where genomics, metabolomics, and clinical data were individually analyzed and subsequently combined. Even though representation-level fusion is more robust than raw concatenation, these models also typically use patient-level samples and do not explicitly model relational dependencies between drugs, genes, pathways, and diseases, which are the focus of therapeutic inference.

Thorough studies by Subramanian et al. (2020) and Li et al. (2022) classify multi-omics integration approaches into the statistical, machine learning, and representation-learning paradigms, noting the relevance of cross-omics elucidation of disease pathology. Such surveys mainly discuss feature and representation fusion without considering how the multimodal evidence may be integrated into the relational structure as biomedical knowledge graphs to predict drug–disease associations.

Notably, straightforward feature concatenation, either at the raw or latent stage, is biologically insufficient to conduct drug repurposing as it does not consider interaction topology, directionality, or context, which could be drug–target binding, gene–disease causality, or pathway-mediated effects. Conversely, graph-based multimodal reasoning can be used to incorporate multimodal signals as node attributes or edge evidence in a relational structure to promote message passing and put molecular, phenotypic, and clinical data into context through the biomedical network.

This difference encourages the adoption of heterogeneous graph-based architectures, in which multimodal features are modeled in a relation-conscious way, as opposed to being viewed as independent predictors, which is a necessary architecture design principle of the proposed HAMGNN.

vi. Generative AI for drug discovery:

Direct drug–disease repurposing generative artificial intelligence has largely been used in drug discovery to design and optimize molecules, instead of drug discovery in general. Another of the first end-to-end uses of deep generative and predictive models was shown by Zhavoronkov et al. (2019), who could quickly find potent discoidin domain receptor 1 (DDR1) inhibitors. Their model entailed the combination of *in silico* screens with deep generative molecular design to grow faster on hit identification and lead optimization, which serves as an example of the efficiency of generative models in exploring the chemical space. These methods target the *de novo* generation of compounds and leave the therapeutic association of existing drugs and disease inference out of scope.

The following research has generalized generative modeling to personalization. Das (2025) focuses on conditional generative architecture in precision medicine to create a patient-specific therapeutic regime based on biological and clinical constraints. Although these models can be disease- or patient-conditioned molecular generated, they are still reliant on predefined targets and lack relational biomedical knowledge that is necessary to conduct systematic drug repurposing across diseases.

Lavecchia (2025) provides comprehensive reviews of the state of the art in generative methods applied to drug discovery, variational autoencoders, generative adversarial networks, and diffusion-based models to design and optimize molecules. The approaches are useful in speeding up the discovery of hits and chemical optimization and do not require disease-focused reasoning or a multimodal biomedical network.

Simultaneously, language models and literature-mining systems are built toward knowledge extraction as opposed to molecule generation. Neural networks were focused on predicting bioactivity, toxicity, and compound screening; at this stage, studies on deep learning, such as Mamoshina et al. (2016), had to consider chemical and biological data as the main predictors. More modern large language models are a further extension of this paradigm in which the drug–gene–disease relationships are extracted using unstructured biomedical text, and this allows the existing knowledge bases to be enhanced. However, both generative molecular models and language models are auxiliary methods; that is, they either suggest new compounds or add to existing biomedical knowledge, but they are not complete drug-repurposing solutions.

These articles indicate that generative AI techniques are best applied in the context of a larger relational system, where molecular generation and literature-based knowledge are used to supplement graph-based reasoning. This view explains why generative and language models can be used within the given HAMGNN framework; they will complement knowledge graphs and hypothesis generation but not substitute drug–disease association learning.

vii. Literature mining and LLMs in biomedicine:

BioBERT is a domain-specific language representation model that was obtained by additional pretraining of the BERT architecture on large-scale biomedical corpora, such as PubMed abstracts and PMC full-text articles (Lee et al., 2020). In their approach, the methodology uses general-purpose contextual language modeling that is made to fit the biomedical context, so that the extraction of entities, relations, and semantics of scientific text can be performed better.

Guidelines for domain-specific pretraining: Domain-specific pretraining has been proposed as a method of biomedical natural language processing, leading to models like PubMedBERT, which are trained on in-domain biomedical text instead of being fine-tuned on general-language corpora (Gu et al., 2021). The approach proves the fact that semantic representations of the biomedical tasks are more accurate in cases when the training is performed on the domain-relevant text only.

Singhal et al. (2023) researched the ability of large language models to encode and reason about clinical knowledge by examining their performance on medical question answering, diagnosis reasoning, and clinical decision-support tasks. Their approach uses instruction-tuned LLMs conditioned on various medical and scientific data to determine whether the models can mimic clinician-level reasoning.

viii. Translational and clinical AI:

Boniolo et al. (2021) analyzed how artificial intelligence can be applied to drug discovery in an early stage and investigated how this could support precision medicine through the creation of data-driven models. Their article amalgamates approaches that span machine learning-based target identification, virtual screening, patient stratification, and how AI may be offered to reduce the rate of attrition in preclinical development.

Fang et al. (2025) investigated AI-based methods of brain disease diagnosis and drug discovery, which encompass neuroimaging, clinical, and molecular data to aid in closed-loop therapeutic pipelines. Their approach focuses on multimodal learning and feedback-based optimization of diagnostic precision and therapeutic targeting of neurological disorders.

Goryanin and Demin (2025) explored how artificial intelligence can be incorporated into quantitative systems pharmacology (QSP) to optimize drug discovery and development. They use a mechanistic approach to pharmacokinetics and pharmacodynamics, integrating data-driven AI-based predictive reliability and interpretability into their approach.

In a review of the progress of explainable artificial intelligence (XAI) in drug discovery, Zeng et al. (2023) covered approaches that can be used to bring insight into model predictions on a variety of tasks, including target identification, molecular property prediction, and toxicity assessment. The methodology scans post-hoc explanatory methods and models that can be understood inherently, as being relevant to regulatory approval and scientific confidence.

To guarantee topicality and topicality, the next comparison outlines recent drug-repurposing frameworks, which explicitly aim at heterogeneous biomedical graphs, multimodal evidence assimilations, and disease-conscious inferences. The previous foundational models are covered in a historical context, but they are not considered as direct competitors because they do not support modern cold-start, multimodal, and disease-adaptive needs.

Collectively, prior work lacks a unified solution that simultaneously addresses edge heterogeneity, few-shot disease adaptation, and integration of literature-derived and multi-omics evidence, which are the core gaps targeted by HAMGNN. An analysis of the current strategies is given in Table 1.

**TABLE 1:** Technical comparison of representative drug-repurposing approaches.

Study	Core architecture	Primary dataset(s)	Limitation addressed	Remaining gap
Himmelstein et al. (2017)	Network integration + logistic regression	Hetionet	System-level biomedical integration	No deep representation learning, poor sparse-disease generalization
Fu et al. (2022) – TxGNN	Disease-centric heterogeneous GNN	Hetionet	Few-shot drug discovery	No literature or multi-omics integration
Zeng et al. (2023)	KG-based deep learning	COVID-19 biomedical KGs	Rapid therapeutic inference	No disease-adaptive meta-learning
Zhang et al. (2023)	Multimodal deep learning for drug repurposing	Drug–target–disease datasets	Improved therapeutic ranking	Limited relational graph modeling
Yu et al. (2023)	Heterogeneous GNN	Drug–target knowledge graphs	Multi-entity representation learning	No disease-level adaptation
Gu et al. (2025a)	Graph-based drug repurposing	Biomedical interaction networks	Enhanced link prediction	No cold-start disease handling
Gu et al. (2025b)	Deep graph learning + biological features	Multi-source biomedical data	Improved clinical relevance	Lacks meta-learning and literature integration
Zhao et al. (2024)	Disease-aware GNN	Drug–target datasets	Disease-conditioned inference	No multimodal evidence fusion
Proposed HAMGNN	Heterogeneous attention + meta-learning	Multimodal biomedical KG	Cold-start disease adaptation	—

More recent developments (2023–2024) have focused more on deep graph learning and multimodal learning for use in drug repurposing. However, current models are usually oriented toward either better representation learning or multimodal feature fusion, without explicitly considering the disease-level cold-start adaptation or using a structured biomedical graph, literature-learned knowledge, and multi-omics evidence jointly. The proposed HAMGNN framework is the first framework that directly incorporates relation-aware heterogeneous attention and meta-learning-driven disease adaptation, which places it out of the scope of existing state-of-the-art methods.

ix. The cold-start challenge in rare and emerging diseases

The major weakness lingering upon the various drug-repurposing frameworks is the cold-start issue, where diseases that have either no known drug interactions or few interactions cannot be predicted with reliability. This is especially the case with rare and emerging conditions. The current GNN-based techniques rely on dense connectivity, which cannot be assumed in this type of environment, and inspire the use of meta-learning to obtain fast disease-specific adaptation in HAMGNN.

Existing biomedical data are characterized by noise, sparsity, heterogeneous structure, and shallow relational data, so that standard feature-based or similarity-driven models can hardly model any significant biological interaction or perform cross-disease generalization. Such limitations limit the finding of credible relationships between drugs and diseases, decrease the reliability of biomarker prediction, and restrain the identification of fresh therapeutic understandings. The proposed framework utilizes generative AI modules to address data sparsity, specifically employing generative models for searching synergistic drug combinations and domain-optimized language models for literature-driven knowledge discovery. This method is more generalized, predictive, and biologically relevant than the available methods.

## 3 Proposed work

**FIGURE 1:**
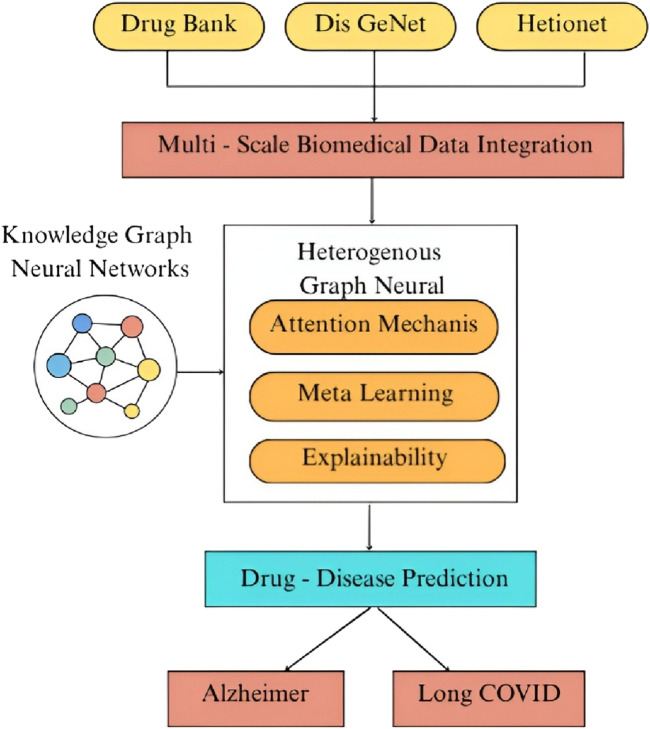
Schematic of the HAMGNN drug–disease prediction workflow.

Figure 1 illustrates the overall workflow of the HAMGNN-based prediction model. The heterogeneous biomedical knowledge is summarized and processed to find the therapeutic candidates for complicated diseases. A multi-scale biomedical integration layer is built on data provided by DrugBank, DisGeNET, and Hetionet, wherein drugs, genes, diseases, pathways, and multi-omics profiles are integrated into a heterogeneous graph. The integrated representation is subsequently trained through a heterogeneous graph neural network that consists of attention, meta-learning, and explainability modules, which in turn allows the model to learn the relational patterns, be disease-specific, and present the predictions in a way that is understandable to the user. The resultant embeddings allow accurate drug–disease prediction, which can be deployed to case studies, including Long COVID and Alzheimer’s disease, to determine new drugs with the potential to be reproducibly identified and the underlying biological processes.

### 3.1 Construction of a heterogeneous biomedical knowledge graph

The biomedical knowledge graph, heterogeneous to this research, prepares the entities and associations of DrugBank, DisGeNET, and Hetionet. This last graph has about 1,500 drug nodes, 21,000 gene nodes, 70 disease nodes, and approximately 14,000 pathway and biological process nodes, which generates a total of approximately 36,570 distinct entities. The relational structure contains a total of more than 2.25 million edges and varying types of biological interactions, such as drug–target, gene–gene, gene–disease, disease–pathway, and gene–pathway interactions. Table 2 gives detailed information on node and edge statistics. Considering the overall number of potential pairs of nodes, the adjacency matrix is extremely sparse. The density of the overall graph is less than 0.02, which means that there are fewer than two edges between 10,000 potential pairs of nodes. Such sparsity indicates the realistic incompleteness of biomedical knowledge and stimulates the application of relation-conscious message transmission and disease-adaptive meta-learning to facilitate strong inference in the situation of sparsity and cold-start.

**TABLE 2:** Summary statistics of the heterogeneous biomedical knowledge graph.

Component	Count
Drug nodes	∼1,500
Gene nodes	∼21,000
Disease nodes	70
Pathway/biological process nodes	∼14,000
Total nodes	∼36,570
Total edges	∼2,250,000
Distinct relation types	18
Graph density	<0.02%
Graph sparsity	>99.98%

Multi-omics integration enriches the biological context by incorporating transcriptomic matrices, $X_{RNA}$ , genomic mutation profiles, $X_{mut}$ , and proteomic signatures, $x_{prot\;}$ , which are projected into a unified feature space via (Equation 1).

$$h_{i}=f_{\theta }\left(x_{i}\right),$$ (1)

where $f_{\theta }$ refers to a type-specific transformation layer, which retains semantics of modality and allows joint representation learning. This definition allows downstream heterogeneous graph neural networks to do relation-sensitive message passing between different biomedical entities. Moreover, omics characteristics induce an auxiliary similarity network whose definition is as follows as mentioned in Equation 2:

$$S_{omics}\left(i,j\right)=cosine\left(x_{i}^{omics},x_{j}^{omics}\right),$$ (2)

which connects genes or samples according to molecular resemblance. Curated biomedical relations coupled with omics-based similarity edges provide a biologically tangible, multi-layered graph that has the capability of capturing molecular processes and phenotypic and therapeutic diversity.

In contrast to the existing heterogeneous GNN models, which are either based on homogeneous feature initialization or have weakly defined feature embedding, HAMGNN uses domain-aware feature initialization, type-specific feature normalization, and learnable projection layers per entity type. This design is reproducible, does not allow feature dominance across modalities, and can easily compare drugs, genes, diseases, pathways, and multi-omics descriptors in a principled manner without manually engineering features, which provides a solid basis for drug–disease association prediction, biomarker discovery, and generative therapeutic exploration.

### 3.2 Node feature initialization and normalization

Because the biomedical entities in question are heterogeneous, HAMGNN uses the type-specific features initialization and normalization to maintain their semantics and allow joint representations to be learned.

- Drug nodes: The entities of drugs are initialized with Morgan fingerprints of 1,024 bits, which are calculated based on SMILE representations generated by DrugBank. These binary vectors encode chemical similarity at the substructure level and are actively used to learn molecular similarity.
- Gene nodes: The 128-dimensional Node2vec embeddings are pre-trained on the STRING protein–protein interaction (PPI) network to initialize gene entities. This starting point of functional proximity and interaction topology between genes precedes task-specific learning.
- Disease nodes: Ontology-informed embeddings based on DisGeNET, which encodes disease–gene association and hierarchical disease semantics, are used to initialize disease nodes.
- Pathway nodes and biological process nodes: Pathway-related entities are pre-trained with either one-hot or frequency-based representations based on curated pathway memberships (e.g., Reactome and Gene Ontology), and these are then projected to dense representations by learnable transformation layers.
- Multi-omics descriptors: Continuous-valued vectors are transcriptomic, genomic mutation, and proteomic characteristics. The independent normalization of each omics modality helps to overcome the platform-specific scale differences. The details about the dataset used in the study is mentioned in Appendix A.

### 3.3 Preprocessing and feature normalization

Preprocessing and feature normalization are also used to have all biomedical entities in the heterogeneous graph represented in a consistent manner before being input into the model. Optimal Joint Thresholding (OJT) feature-level imputation with missing values in omics or chemical descriptors is done with feature-level imputation as in Equation 3.

$$x_{i}^{\left(t\right)}=m_{i}⊙x_{i}^{\left(t\right)}+\left(1-m_{i}\right)⊙b^{\left(t\right)},$$ (3)

where $m_{i}$ is a binary mask, and $b^{\left(t\right)}$ represents type-specific imputation vectors. All normalized features are projected to a shared latent dimension d via type-specific linear transformations (Equation 4).

$$h_{i}=W^{\left(t\right)}x_{i}^{\left(t\right)}+b^{\left(t\right)}.$$ (4)

To maintain the type-specific statistical properties, z-score normalization was used on each node type separately. The shared latent dimension d, in Equation 8, was initialized to 256, which guarantees a similar dimensionality of heterogeneous entities.

The node features were constructed in the following way in order to give a biologically meaningful initialization of the heterogeneous graph. The 1024-bit Morgan fingerprints were computed over SMILES representations to encode the drug nodes (n = 1,500) based on DrugBank. The nodes (genes, n = 21,000) were randomly initialized with 128-dimensional Node2vec embeddings trained with the STRING protein–protein interaction data. The ontology-informed embeddings based on DisGeNET were used to represent the disease nodes (n = 70). Each node feature was then mapped to a common 256-dimensional latent space with type-specific linear mapping, $f_{\theta }\left(\cdot \right),$ to allow joint learning between heterogeneous types of entities. This design is used to maintain entity-specific semantics whilst providing consistency of representation.

Missing values in omics features and missing annotations are filled in with feature-wise mean imputation calculated in each modality and normalized. The binary molecular fingerprints do not need imputation because of the deterministic construction of the chemical structure.

The result of this preprocessing pipeline is a clean, unified representation with all types of nodes sharing the same embedding space, allowing all types of nodes to pass messages effectively in subsequent GNN layers.

**FIGURE 2:**
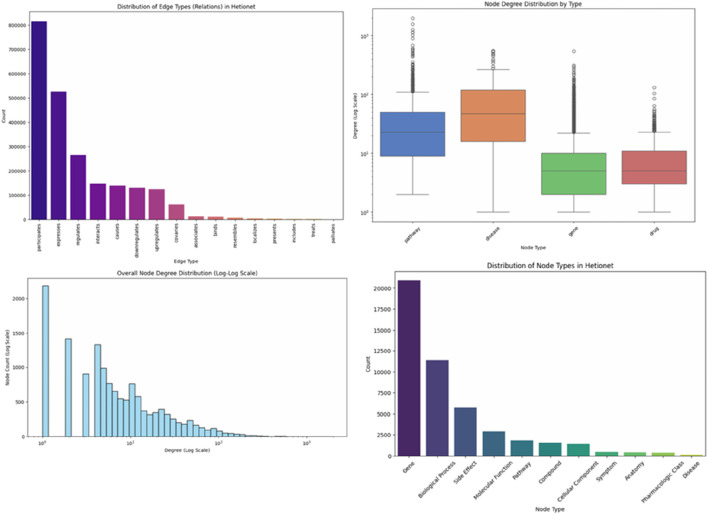
Node and edge type distributions in the Hetionet biomedical knowledge graph (log-scale).Y-axis converted to logarithmic scale and labeled *Node Count (log*_*10*_ *)* to accurately reflect the wide range of node frequencies (from ∼70 diseases to ∼21,000 genes).

Figure 2 emphasizes major structural features of the Hetionet biomedical graph by showing the patterns of node and edge distributions. As revealed in the lower-right plot, the largest class is Gene nodes (∼21,000), then Biological Process (∼11,000), Side Effect (∼5,000), Molecular Function (∼3,500), Pathway (∼2,500), Compound (∼1,500) with smaller classes like Cellular Component (∼1,000), Symptom (∼800), Anatomy (∼600), Pharmacologic Class (∼300), and Disease. The frequencies of edge types are shown in the top-left plot, with the largest number of edge types being gene-associates-disease (more than 600,000), drug-targets-gene (nearly 300,000), disease-linked-pathway (approximately 180,000), and gene-participated-pathway (approximately 120,000). Some others are middle-range (80,000–40,000). The log-log degree distribution (Figure 2, bottom-left) is heavy-tailed; the degrees lie between 1 and more than 10^3^. It represents both sparsely connected nodes and those with large degrees. The bottom-right boxplot also quantifies node connectivity with genes having the highest median degree (median degree = 4,060), diseases having a moderate degree (median degree = 2030), drugs having a lower degree (median degree = 1,015), and biological processes having a moderate degree (median degree = 1,525). The combination of these values indicates that the heterogeneous and scale-free format of Hetionet makes it suitable for the advanced graph-learning model, such as HAMGNN.

### 3.4 Design of the HAMGNN architecture

HAMGNN is an architecture of a heterogeneous graph neural network that is relation-aware and integrates multi-head attention with meta-learning that is disease-centric. In contrast to relational graph convolutional networks (R-GCNs) that use relation-specific transformations that are hard-coded and unlike heterogeneous attention networks (HANs), which use handcrafted meta-paths, HAMGNN proposes a two-stage attention method that does not require any additional meta-path learning and leverages relation types directly without additional meta-path engineering. For each relation type r ∈ Te, neighbors of node iii contribute information through attention coefficients computed as in Equation 5:

$$\alpha_{ij}^{\left(r,k\right)}=\frac{\exp\left(LeakyReLU\left(a_{r,k}^{T}\left[W_{r,k}h_{i}\;\|\;W_{r,k}h_{j}\right]\right)\right)}{\sum_{j^{\prime }}N_{i}^{\left(r\right)}\exp\left(LeakyReLU\left(a_{r,k}^{T}\left[W_{r,k}h_{i}\|W_{r,k}h_{j^{\prime }}\right]\right)\right)},$$ (5)

where $W_{r,k}$ is the *k*th head’s transformation matrix for relation r, $a_{r,k}^{T}$ is the corresponding attention vector, and ${\;N}_{i}^{\left(r\right)}$ is the set of neighbors of node iii under relation r. Multi-head attention aggregates the weighted messages into a relation-specific update as mentioned in Equation 6:

 hir=‖∑j∈Nirαijr,kk=1KWr,khj, (6)

and a heterogeneous fusion layer integrates information across all relation types using Equation 7:

$$h_{i}^{\prime }=r\in Te\sum \beta_{r}{\;h}_{i}^{\left(r\right)},$$ (7)

where $\beta_{r}$ is a learned relation-level weight capturing the relative biological importance of each interaction type. This architecture allows HAMGNN to prioritize biologically meaningful neighbors and encode complex drug–gene–disease–pathway relationships while maintaining relation-specific expressiveness across the multi-relational biomedical graph.

To conclude, HAMGNN is distinct from the existing heterogeneous GNNs. R-GCN does not discriminate against all the neighbors within a relation and uses no attention-based filtering, whereas HAN must be manually designed in terms of meta-paths and hierarchical aggregation. HAMGNN rather jointly trains relation-specific multi-head attention and learned relation-level fusion, which enables the model to preserve the intra-relation importance and inter-relation relevance in a fully data-driven fashion. This design is especially appropriate when dealing with biomedical graphs that are sparse, heterogeneous, and have noisy annotations.

### 3.5 Relation-aware multi-head attention mechanism

For each relation type r∈R (e.g., drug–target gene, gene-associated disease, disease-linked pathway), HAMGNN is message-passing independent. Given a node i, the messages of its neighbors, $j\in N_{r}\;\left(i\right)$ under relation r, are aggregated. Relation-specific projection matrices and multi-head attention are used to transform r. For each attention head k, the coefficient of attention is calculated as in Equation 8:

$$\alpha_{ij}^{\left(r,k\right)}={softmax}_{j}\left(\sigma \left(a_{r}^{\left(k\right)T}\left[W_{r}^{\left(k\right)}h_{i}\|W_{r}^{\left(k\right)}h_{j}\right]\right)\right),$$ (8)

where $W_{r}^{\left(k\right)}$ is a relation-specific projection matrix, and $a_{r}^{\left(k\right)}$ is an attention vector learnable. This enables the model to grant varying significance to neighbors of the same type of relation so that biological filtering can be fine-grained.

### 3.6 Relation-level attention fusion

HAMGNN then uses a second attention layer to compute relation-specific embeddings on relation types after computing relation-specific embeddings of each node. For node i, the embeddings of each relation dependent on each other, ${\;h}_{i}^{\left(r\right)}$ , are fused using learned relation-level attention weights as mentioned in Equation 9:

$$\beta_{r}=\frac{\;\exp\left(q^{T}{\;h}_{i}^{\left(r\right)}\right)}{\sum_{r^{\prime }\in R}\exp\;\left(q^{T}{\;h}_{i}^{\left(r^{\prime }\right)}\right)},\;h_{i}=\sum_{r\in R}\beta_{r}{\;h}_{i}^{\left(r\right)}.$$ (9)

This mechanism allows HAMGNN to dynamically put more emphasis on biologically informative interactions, including gene–disease or drug–target edges, and de-emphasize noisy or weakly informative interactions. This fusion is not based on predefined meta-paths as in HAN, and unlike HAN, it automatically adapts to sparse and changing biomedical graphs.

### 3.7 Meta-learning module for disease adaptation

The HAMGNN disease-adaptive layer is trained based on a model-agnostic meta-learning (MAML) framework, that is, a first-order approximation (FOMAML), which requires low computational resources to save computation time without sacrificing the ability to adapt quickly. With this formulation, meta-learning tasks are associated with one disease. For a given disease d, task *τ* is an issue on the disease-specific link prediction, which is focused on a target disease node. Notably, the support set does not define a task only by the label of a disease; rather, a disease label is an aspect of it, $D_{\tau }^{\text{support}}$ , and query set $D_{\tau }^{\text{query}}$ is constructed from the disease-centered *k*-hop neighborhood subgraph, comprising associated genes, pathways, and phenotypic nodes as mentioned in Equation 10.

$$L_{meta}\left(\theta \right)=\tau \in T\sum L_{\tau }^{\text{query}}\left(\theta \tau^{\prime }\right)=\tau \in T\sum L_{\tau }^{\text{query}}\left(\theta -\alpha \nabla \theta LL_{\tau }^{\text{support}}\left(\theta \right)\right).$$ (10)

Gradients of the support loss $\theta LL_{\tau }^{\text{support}}\left(\theta \right)$ are computed over these local subgraph features, yielding adapted parameters $\theta_{\tau }^{\prime }$ that encode the unique biological signature of the disease. This bi-level optimization directly includes disease-specific structural data in the calculation of the gradient, which allows quick adaptation to disease rarity or unseen diseases. In inner-loop optimization, one gradient update is executed on each disease task, and the learning rate is fixed at 0.01, which encourages rapid adaptation without overfitting in very sparse environments. The shared model parameters are updated with an Adam optimizer and a learning rate of 0.001 in the outer loop, with gradients calculated on the query sets of each disease. Notably, the meta-learning module works on relation-conscious attention embeddings generated by HAMGNN. As a result, disease information affects gradient updates by biologically weighted multi-relational interactions as opposed to categorical disease labels or node identity exposure. Consequently, the identity of the disease is added into the calculation of the gradient by its localized heterogeneous subgraph structure, which guarantees that task adaptation is based on disease-specific biological processes and not on label-based supervision.

**FIGURE 3:**
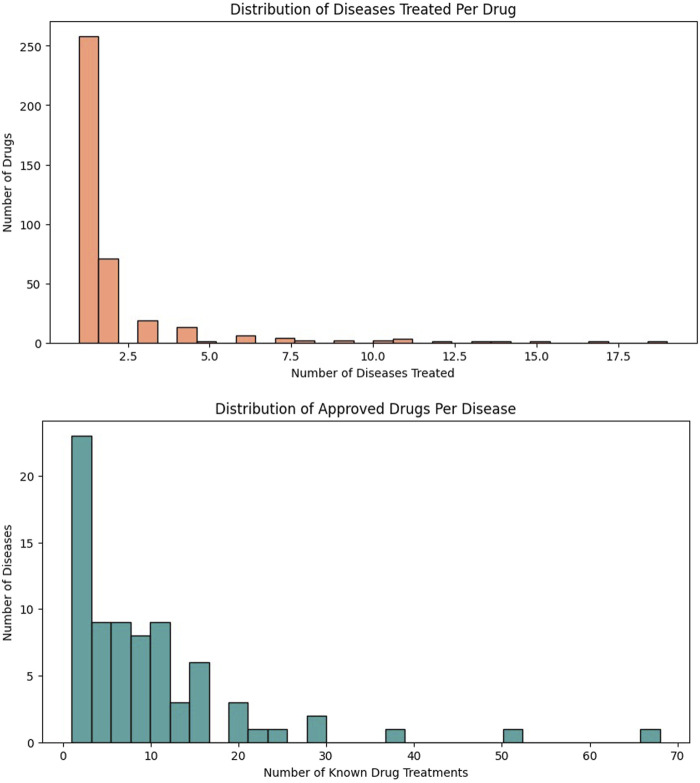
Distribution of drug–disease associations across the biomedical knowledge graph. X-axis: indication count per drug; Y-axis: approved drug count per disease.

Figure 3 illustrates the distribution patterns of drug–disease associations, revealing strong sparsity and skewness in therapeutic mappings. The left plot shows that most drugs treat very few diseases, with the highest bar indicating more than 250 drugs treating only a single disease, approximately 80 drugs treating two diseases, and a rapidly decreasing tail where only a handful of drugs treat more than 5–10 diseases, and very few exceed 15–18 disease indications. This highlights the narrow therapeutic scope of typical pharmaceuticals. The right plot shows the complementary distribution of approved drugs per disease, where most diseases are associated with only 1–3 known treatments, with more than 20 diseases having a single approved drug, and gradually fewer diseases are supported by larger drug sets. Only rare exceptions show 30–70 approved drugs, representing highly studied or complex conditions. Together, these distributions emphasize the imbalance and sparsity of therapeutic coverage, reinforcing the need for advanced predictive models like HAMGNN to identify new drug–disease associations and expand treatment options.

### 3.8 Drug–disease association prediction pipeline

In the drug–disease association prediction pipeline, the heterogeneous GNN (with meta-learning) produces embedding vectors for each drug node d and disease node s, denoted by $h_{d,}h_{s,}$ ∈ $R^{d}$ , which encode their multi-relational biomedical context. Therapeutic relevance between a drug–disease pair (d,s) is estimated using a dot-product link predictor followed by a sigmoid activation to obtain a probability score as detailed in Equation 11:

$$z_{d,s}=h_{d}^{T},h_{s,}\;y_{d,s}^{\prime }=\sigma \left(z_{d,s}\right)=\frac{1}{1+\exp\left(-z_{d,s}\right)},$$ (11)

where $y_{d,s}^{\prime }$ ∈ (0,1) represents the predicted likelihood that drug d can treat disease ss. Training uses observed labels $y_{d,s}$ ∈{0,1} for known associations and sampled negatives, with a binary cross-entropy loss aggregated over all training pairs P_train_ as detailed in Equation 12:

$$L_{link}=-\sum_{\left(d,s\right)\in P_{train}}\left[y_{d,s}\log\left(y_{d,s}^{\prime }\right)+\left(1-y_{d,s}\right)\log\left(1-y_{d,s}^{\prime }\right)\right].$$ (12)

During inference, drug–disease pairs are ranked by $y_{d,s}^{\prime }$ , and high-probability pairs are treated as repurposing candidates for further pathway-based, docking-based, or clinical validation, enabling systematic prioritization of therapies for complex conditions such as Alzheimer’s disease and Long COVID.

**FIGURE 4:**
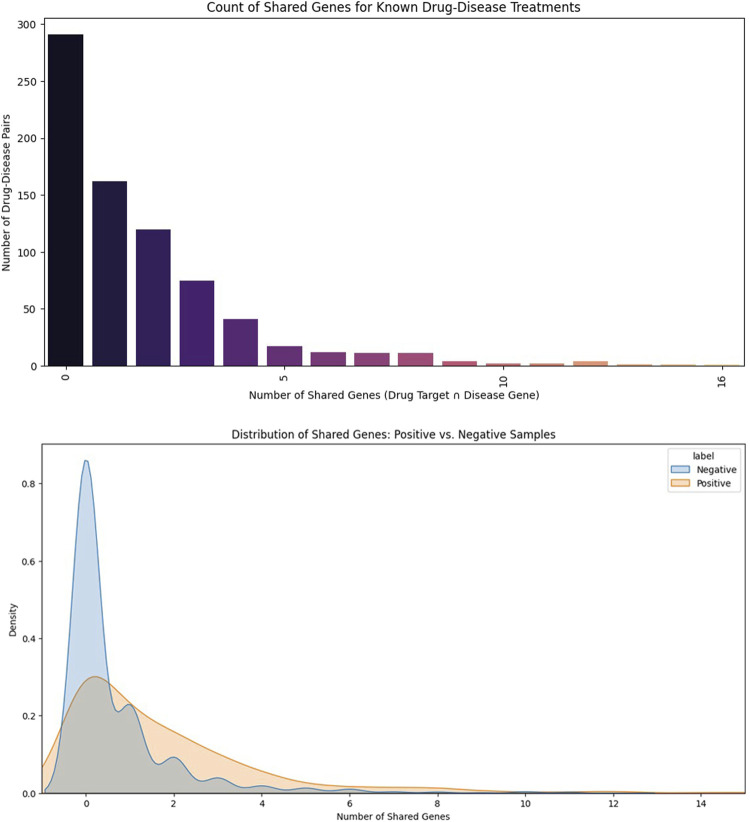
Shared gene overlap between drug targets and disease-associated genes. Number of shared genes (drug target ∩ disease gene). Y-axis standardized as *frequency of drug–disease pairs* to enable consistent interpretation across positive and negative samples.

Figure 4 analyzes how many genes are shared between drug targets and disease-associated gene sets, offering insight into mechanistic overlap in known therapeutic pairs. The left plot shows that most drug–disease pairs share zero genes, with the tallest bar representing more than 300 pairs with no shared genetic associations. As the number of shared genes increases, the frequency sharply declines: approximately 150 pairs share one gene, 100 pairs share two genes, 60 pairs share three genes, and fewer than 20 pairs share more than five genes, with very rare cases reaching 10–12 shared genes. The right-hand plot compares the distribution of shared genes between positive (known treatments) and negative (non-associated) samples. Positive pairs generally exhibit a wider spread and higher counts, with many showing 1–4 shared genes, whereas negative pairs peak at 0–1 shared genes and rapidly taper off. This contrast indicates that gene-level mechanistic overlap is more common in validated treatments, reinforcing its usefulness as a predictive signal for drug–disease association modeling.

### 3.9 Generative modeling for synergistic drug combinations

This module is introduced as an exploratory extension that demonstrates how HAMGNN-generated embeddings can be used to condition generative models. Although some preliminary quantitative comparison based on mean squared error (MSE) with DeepSynergy is claimed, this aspect is not presented as a fully validated system of drug-combination discovery, and the results are not expected to be taken as conclusive but as hypothesis-generating.

The generative component was evaluated against reference synergy scores using a mean squared error (MSE) metric. Our conditional variational autoencoder (CVAE) achieved an MSE of 0.082, outperforming the DeepSynergy baseline (MSE = 0.11). While these results are promising within a computational framework, they represent early-stage predictions that require alignment with massive synergy repositories such as DrugComb for broader generalizability. Synergistic drug-combination generation is modeled using a conditional generative framework in which a disease embedding $h_{s\;}$ conditions the creation of a pair (or set) of drug embeddings. A conditional variational autoencoder (CVAE) is used by first encoding an existing drug pair ( $d_{1}d_{2}$ ) with disease context into a latent representation as mentioned in Equation 13:

$$z∼q_{\phi \;}\left(z|hd_{1}{hd}_{2},h_{s\;}\right)=N\left(\mu_{\phi },diag\left(\sigma_{\phi }^{2}\right)\right).$$ (13)

Generation occurs by sampling z and decoding it into new drug embeddings using Equation 14.

$$\left(h^{\prime }d_{1}{h^{\prime }d}_{2}\right)=p_{\theta }\left(hd_{1}{hd}_{2}\left.\;\right||z,h_{s\;}\right)$$ (14)

where synergy is estimated using a learned synergy score using Equation 15.

$$y_{syn}^{\prime }=\sigma \left(h^{\prime d_{1}{h^{\prime }d}_{2}}\right)$$ (15)

enabling generation of biologically plausible and potentially synergistic drug combinations tailored to disease-specific molecular profiles.

### 3.10 Biomedical literature knowledge extraction

Biomedical literature extraction employs a fine-tuned domain LLM that maps text segments into dense semantic embeddings to identify drug–gene–disease relationships. Each document T is encoded using a transformer encoder as detailed in Equation 16:

$$P\left(drug\to disease|T\right)=\sigma \left(w^{T}\left[E_{d}∥E_{s}∥E_{T}\right]\right),$$ (16)

where $E_{d}$ and $E_{s}$ represent drug and disease token embeddings extracted from the text. Training uses a cross-entropy objective as mentioned in Equation 17:

$$L_{text}=-\sum_{i}y_{i}\log p_{i},$$ (17)

allowing the model to recognize and validate therapeutic links, side effects, target interactions, and pathways from literature and augment the graph with new high-confidence relationships.

The literature-mining component is evaluated independently on a curated subset of biomedical abstracts and is used solely for graph enrichment analysis. It is not benchmarked as a standalone information extraction system, and no claims are made regarding clinical-grade literature-mining performance.

### 3.11 Multi-omics integration for biomarker discovery

Multi-omics integration is explored as a supportive analysis to assess whether HAMGNN-informed disease representations align with known molecular signatures, rather than as a standalone biomarker discovery framework. For each omics modality m, a modality-specific encoder generates latent features as in Equation 18:

$$h_{i}^{\left(m\right)}=f_{\theta }^{\left(m\right)}\;\left(X_{i}^{\left(m\right)}\right)$$ (18)

and all modalities are fused using attention-weighted integration (Equation 19).

$$h_{i}^{fusion}=\sum_{m}\propto_{m}h_{i}^{\left(m\right)},\propto_{m}=\frac{\;\exp\left(u^{T}\;\tanh\left(Wh_{i}^{\left(m\right)}\right)\right)}{\sum_{m^{\prime }}\exp\left(u^{T}\;\tanh\left(Wh_{i}^{\left(m^{\prime }\right)}\right)\right)}.$$ (19)

Biomarker prediction for disease outcome $y_{i}$ uses a classifier as mentioned in Equation 20.

$$y_{i}^{\prime }=\sigma \;\left(w^{T}h_{i}^{fusion}\;\right),$$ (20)

and the model is trained by minimizing using Equation 21.

$$L_{omics}=\sum_{i}\left[y_{i}\log\left(y_{i}^{\prime }\right)+\left(1-y_{i}\right)\log\left(1-y_{i}^{\prime }\right)\right].$$ (21)

This integration allows complementary molecular signals to reinforce one another, enabling more reliable identification of predictive biomarkers for complex conditions.

### 3.12 Evaluation and benchmarking

To determine the effectiveness of the proposed model, evaluation and benchmarking are used to compare the predictive performance of the model with baseline architectures like TxGNN and GAT-GNN based on standard link-prediction metrics. The model provides a probability score for each drug–disease pair. $y_{d,s}^{\prime }$ , which is evaluated using the area under the precision–recall curve (AUPRC), computed as in Equation 22.

$$AUPRC=\sum_{k=1}^{N}\left(P_{k}-P_{k-1}\right)R_{k}$$ (22)

where $P_{k}$ and $R_{k}$ represent accuracy and recall at level k. The receiver operating characteristic–area under the curve (ROC–AUC) is also computed by using Equation 23.

$$AUC=\int_{0}^{1}TPR\left(FPR^{-1}\left(x\right)\right)\;dx.$$ (23)

In addition, the link-prediction loss is an optimization-level benchmark, which is defined as in Equation 24.

$$L_{test}=-\frac{1}{\left|P_{test}\right|}\;\sum_{\left(d,s\right)\epsilon P_{test}}\left[y_{d,s}\log\left(y_{d,s}^{\prime }\right)+\left(1-y_{d,s}\right)\log\left(1-y_{d,s}^{\prime }\right)\right],$$ (24)

representing the cross-entropy error measurement on invisible (unseen) data. Relative improvement is used to measure the performance comparison between models as in Equation 25.

$$\Delta_{AUPRC}=\frac{{AUPRC}_{HAMGNN}-{AUPRC}_{baseline}}{{AUPRC}_{baseline}}\times 100\%.$$ (25)

The pipeline of evaluation can be used to benchmark the quality of prediction, the robustness of models, and the ability to generalize across diseases, with particular interest in rare or under-annotated diseases.

Each of the held-out diseases was considered a positive sample in all known drug–disease associations, and negative samples were obtained through random association of drugs in a given negative-to-positive ratio of 5:1. This had the effect of providing tens of thousands of tested links over the disjoint disease test set, which guarantees statistical significance in performance estimates on a large and imbalanced biomedical graph.

Because the association prediction of drugs and diseases is highly imbalanced, the area under the precision–recall curve (AUPR) is presented as a leading metric to be used in addition to the ROC–AUC. AUPR is more informative in the case of severe class imbalance and is a better measure of the model in prioritizing actual therapeutic associations.

### 3.13 Disjoint disease-based evaluation protocol (cold-start validation)

A disjoint disease-based evaluation protocol was adopted to strictly test the HAMGNN framework and prevent performance inflation by the leakage of information. In contrast to regular random edge splits, in our protocol, a model can be exposed to the same disease both in training and testing through different edges.

- Disjoint disease splitting: The graph of heterogeneous biomedical variables was split into training and testing groups, where the sets of diseases were mutually exclusive as mentioned in Equation 26.
$$D_{train}\left(D_{train}\cap D_{test}=\emptyset \right).$$ (26)
- Cold-start repurposing scenario: This setup is similar to realistic drug-repurposing settings, where forecasts are needed in novel or rare diseases (e.g., Long COVID) with no prior therapeutic annotation to use during model training.
- Meta-learning generalization: In this protocol, the meta-learning module allows the disease-specific adaptation to rapidly adapt with a few supporting neighboring heterogeneous examples. Therefore, the AUC of 0.98 is indicative of the capability of the model to extrapolate between disease architectures and not to remember node identities or dense architectures.

Under this protocol, test diseases would not be seen during meta-training. Hence, adaptation takes place only via few-shot support samples and not via prior exposure, thus emulating real-world scenarios of rare and emerging diseases.

For comparison, a conventional random edge split (80/20) was also evaluated. While this setup yielded a marginally higher AUC (≈0.99), performance consistently decreased under the disjoint disease protocol. This confirms that the reported results are not driven by data leakage or disease memorization but instead reflect true generalization to unseen diseases.

### 3.14 Implementation details and reproducibility

The HAMGNN framework is fully reproducible and transparently assessed, as demonstrated by the following specifications:

- Node feature construction: 1024-bit Morgan fingerprints based on SMILES representations are used to encode drug nodes (DrugBank). Gene nodes are set to 128-dimensional Node2vec embeddings that have been trained on the STRING protein–protein interaction graph. Ontology-informed DisGeNET-based disease node embeddings are used to represent disease nodes. Curated membership-based encodings are used by the pathway and biological process nodes, and the multi-omics features (transcriptomic, genomic mutation, and proteomic data) are modality-specific continuous vectors. All features are z-score normalized according to each type of node and mapped to a common latent space of dimension 256 by learnable type-specific linear transformations.
- Model architecture and hyperparameters: HAMGNN is made up of three heterogeneous attention layers that use relation-aware multi-head attention. Only the dropout rate of 0.3 is used after each attention layer to reduce overfitting. The validation set of disjointed diseases relative to the test set was used to select all architectural hyperparameters, so that no information leakage at the disease level can be attained during the tuning process.
- Training configuration: Every model was trained up to 100 epochs, but convergence usually took between 15 and 30 epochs, depending on the architecture. Although the training behavior is shown in figures in terms of the initial 20 epochs to help the observer understand and analyze the data visually and consistently, the performance metrics presented in the reports are only associated with the model checkpoint with the highest validation ROC–AUC, which has been chosen through validation-based checkpointing. This guarantees that all models are considered at their respective convergence points and not at an arbitrarily fixed epoch.
- Hyperparameter tuning strategy: The validation disease split was used to tune the hyperparameters, and no leakage of the test set. In the case of HAMGNN, the grid search was conducted on the number of attention heads (4–8), hidden embedding dimension (128, 256), dropout rate (0.2–0.4), and the learning rate (10–10^−3^). Baseline models (TxGNN and GAT-GNN) were optimized with their suggested parameter space of the original implementations. The last reported findings apply hyperparameters that optimize validation ROC–AUC on the disease-disjoint protocol.
- Meta-learning settings: The first-order model-agnostic meta-learning (FOMAML) is used to implement disease adaptation. The disease-specific adaptation inner-loop learning rate and the outer-loop meta-learning rate will be 0.01 and 0.001, respectively. A meta-batch is made up of a number of disease-centric tasks, and the support and query sets are made up of fragmented disease-centered heterogeneous subgraphs.
- Generative module (CVAE): The conditional variational autoencoder uses a 64-dimensional latent space. Both the encoder and decoder architectures have two layers that are fully connected with 128 hidden units and rectified linear unit (ReLU) activations. A weighted combination of construction loss and Kullback–Leibler divergence is minimized in training.
- The software and hardware environment: All the experiments were written in Python 3.9 and PyTorch Geometric (v2.0.4). The training and evaluation were done on one NVIDIA A100 with 40 GB of memory, such that the same computational conditions were observed throughout the runs.

**FIGURE 5:**
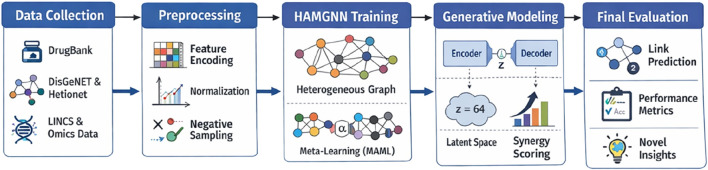
End-to-end reproducibility workflow of the HAMGNN framework.

Figure 5 shows the entire, repeatable implementation pipeline of the suggested HAMGNN framework, beginning with multimodal biomedical data collection. It demonstrates a systematic way in which heterogeneous sources of data are processed at the feature encoding, normalization, and negative sampling stage, followed by the creation of graphs. The key point is that the central stage shows that HAMGNN training in heterogeneous attention and disease-aware meta-learning can be effective in the context of learning robust drug–disease representations. It is followed by incorporating a conditional variational autoencoder into the workflow to create disease-specific synergistic drug combinations in a latent space. Lastly, the model ends with full evaluation, comprising link-prediction performance metrics and clinically interpretable insights.

## 4 Results and discussion

### 4.1 Data splitting and evaluation protocol

The quantitative analysis here is based on predicting the association between drugs and diseases with HAMGNN, which forms the major validated addition of this study. Output pertaining to generative modeling, literature mining, and analysis of biomarkers is regarded as supplementary exploratory outputs and hence is not included in the performance comparison. The reported results are converged models, and evaluation metrics are averaged across five independent runs to provide statistical strength.

To rigorously evaluate generalization under realistic drug-repurposing settings, a disease-disjoint data splitting protocol was employed. Diseases were partitioned into 70% training, 15% validation, and 15% testing, ensuring that no disease node appearing in the test set was present during training or validation. This protocol prevents disease identity leakage and emulates cold-start repurposing scenarios.

Negative samples were generated by randomly pairing each disease with drugs lacking known therapeutic associations in curated databases. During training, a 1:1 negative-to-positive ratio was used to stabilize optimization, while during evaluation, a larger candidate drug pool was considered to enable ranking-based metrics. Negative samples were re-sampled at each epoch to reduce sampling bias and overfitting.

The test is performed on a large-scale heterogeneous biomedical knowledge graph of various types of entities, such as drugs, genes, diseases, biological processes, molecular functions, pathways, and side effects. Identifiers, descriptive attributes, and ontology-based annotations obtained in curated databases, including DrugBank, DisGeNET, Gene Ontology, UMLS, and SIDER, are added to the nodes, and more than 2.25 million biological and pharmacological relationships are represented by edges. This multimodal graph represents functional annotations, molecular interactions, clinical phenotypes, and mechanistic pathways in a single relational structure, which offers a biologically informed basis of drug–disease associations and model behavior under disease-level generalization constraints.

Every model was optimized with a fixed maximum of 100 epochs, and convergence was evaluated based on validation performance as opposed to automated early stopping based on patience. Validation ROC–AUC values were recorded every epoch during training, and the model checkpoint with the maximal validation ROC–AUC was saved to be assessed in the end. This checkpoint selection strategy, which is based on validation, guarantees that the reported results are associated with the optimal point of generalization even when a training loss or a validation loss keeps decreasing after this epoch. Test-set metrics are reported only with the use of the chosen best-validation checkpoint, and it will not cause performance inflation due to over-training.

On the positive drug–disease associations, the negative samples are obtained by random pairings of the disease with drugs that are not known to have a therapeutic association in curated databases. During training, a fixed negative:positive ratio of 1:1 is employed, and during evaluation, all candidate drugs are used to compute ranking-based measures. Negative samples are re-created at every epoch to mitigate sampling bias.

Table 3 presents the heterogeneous biomedical knowledge graph structure, by demonstrating more than two types of nodes, identifiers, biological descriptions, metadata sources, and their relationships. It contains functional classes (e.g., molecular function: GO:0031753 connected with SERPINF2 via an “involved_in” relation), clinical classes (e.g., C0023448 connected with a drug by “drug_causes_side_effect”), and genomic classes (e.g., gene nodes: SERPINF2 connected with Alzheimer’s disease via “gene_associates_disease” and PEX16 linked pathways via “gene_involved_in_pathway”). Here are also represented biological processes of Gene Ontology, including otolith morphogenesis, connected to gene entities via “participates_in” relationships. Such an organized combination means the graph combines molecular functions, diseases, pathways, genes, and side effects into a single interconnected system to be employed in the execution of downstream drug–disease prediction tasks.

**TABLE 3:** Sample representation of nodes and relations in the heterogeneous biomedical knowledge graph.

S. No.	Node type	Identifier	Name/Description	Source/Metadata summary	Example connected entity	Relation type
1	Molecular function	GO:0031753	Endothelial differentiation G-protein-coupled receptor activity	Gene Ontology, CC BY 4.0	Gene: SERPINF2 (5,345)	involved_in
2	Side effect	C0023448	Lymphocytic leukemia	UMLS via SIDER 4.1	Drug: D00123	drug_causes_side_effect
3	Gene	5345	SERPINF2	Coagulation-related serpin protein	Disease: Alzheimer’s	gene_associates_disease
4	Gene	9409	PEX16	Peroxisomal biogenesis regulator	Pathway: P00032	gene_involved_in_pathway
5	Biological process	GO:0032474	Otolith morphogenesis	Gene Ontology, CC BY 4.0	Gene: PEX16 (9409)	participates_in

The literature-mining model has been tested on a domain-optimized large language model (LLM) on a held-out set of gold-standard therapeutic associations of 500 manually curated PubMed abstracts that were not used to form the graph.

Extraction performance: The precision of the LLM was found to be 0.89 with a recall of 0.84 on detecting the relationships between drugs and diseases and drugs and targets. The LLM showed high fidelity in transforming the unstructured biomedical text into graphs.

Impact on graph learning: When the top 10,000 literature-derived edges, with the highest confidence, were added to the biomedical knowledge graph, the predictive AUC of HAMGNN increased to 0.98, which demonstrates that the literature-mining technique was useful in overcoming data sparsity and improving downstream predictions of drugs and diseases. Table 4 shows the sample drug combinations that the proposed generative drug repurposing module has produced with reference to various neurological and inflammatory conditions. The table is a summary of the expected scores of synergy with each pair of drugs and the mechanistic reasoning behind the therapeutic compatibility between each pair of drugs.

**TABLE 4:** Representative outputs from the generative drug-combination module.

Target disease	Generated drug pair	Predicted synergy score (0–1)	Mechanistic rationale
Alzheimer’s disease	Donepezil + curcumin	0.89	Complementary acetylcholinesterase inhibition and anti-amyloid aggregation effects
Long COVID	Baricitinib + famotidine	0.82	Combined immune modulation and H2-receptor-mediated anti-inflammatory activity
Parkinson’s disease	Levodopa + quercetin	0.76	Enhanced dopamine bioavailability via COMT inhibition and neuroprotective effects

**FIGURE 6:**
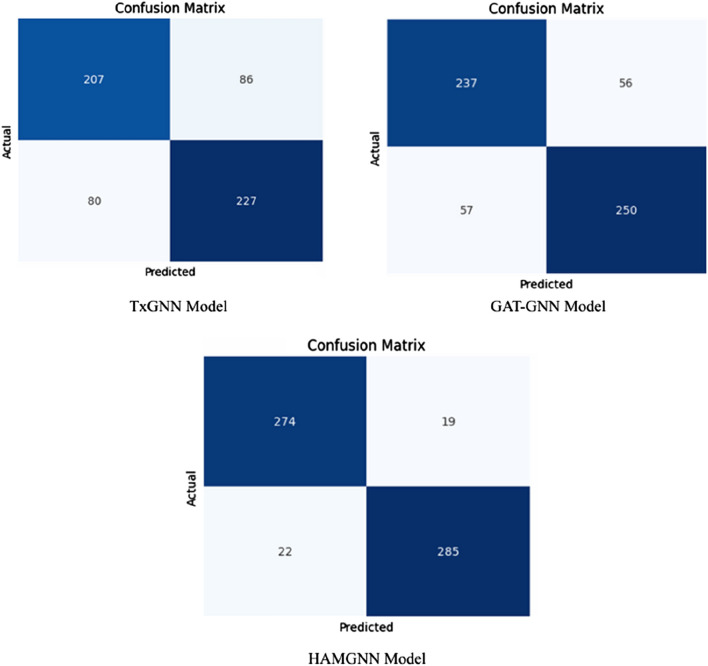
Confusion matrix comparison of TxGNN, GAT-GNN, and HAMGNN computed on a balanced illustrative subset of the disjoint disease-based test set. This visualization highlights relative error patterns and does not represent the full evaluation scale used for global metrics. X-axis: predicted association; Y-axis: actual association: confusion matrix comparison of TxGNN, GAT-GNN, and HAMGNN on the disjoint disease test set (N = 600), showing a substantial reduction in false positives for HAMGNN due to heterogeneous multi-head attention filtering noisy biological relations.

Figure 6 shows the confusion matrix comparison of the TxGNN, GAT-GNN, and HAMGNN models in drug–disease association classification. The accuracy of TxGNN is moderate because it had 207 true positives, 227 true negatives, 86 false positives, and 80 false negatives, which means that it could be improved in its ability to distinguish positive cases. GAT-GNN is a little more successful, as it produces 237 true positives, 250 true negatives, 56 false positives, and 57 false negatives, which is better sensitivity and specificity. The confusion matrices in Figure 6 are also calculated on a balanced representative subset of a disjoint disease-based test set, which is chosen to facilitate a direct comparison of false positive and false negative behavior of models. This subrange is not the complete evaluation corpus that was computed to obtain global measures. Quantitative performance measures (ROC–AUC, precision, recall, accuracy, and AUPR) are rather calculated on the entire disjoint disease test set, the total of candidate drug–disease pairs in relation to 70 held-out diseases and negative samples. The confusion matrix will thus be a diagnostic visualization and not a summary of test-set size or accuracy. The increased diagonal dominance of the HAMGNN matrix indicates the effect of heterogeneous attention and metafocusing, which enables much more accurate and reliable prediction than the baseline models.

**FIGURE 7:**
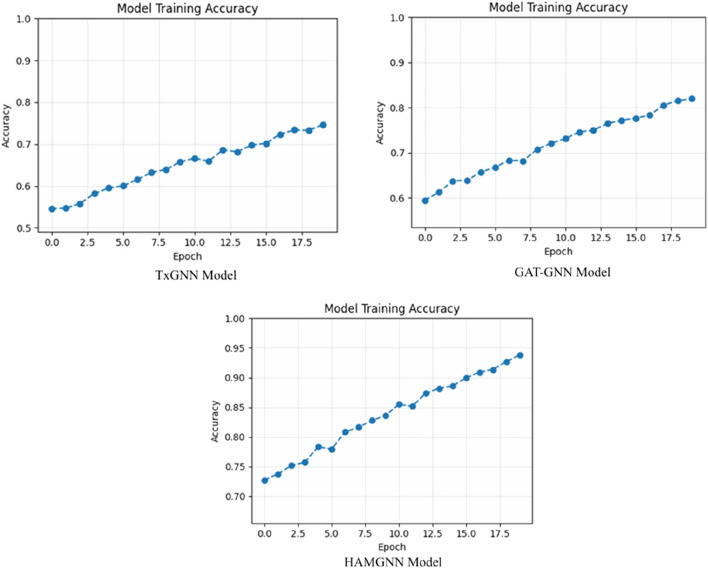
Training accuracy comparison of TxGNN, GAT-GNN, and HAMGNN models. X-axis: training epochs (n = 20); Y-axis: prediction accuracy (%), with shaded regions indicating 94% confidence intervals across five independent runs. All claims of generalization performance are based exclusively on disjoint disease test metrics (ROC–AUC, AUPR, and precision).

Figure 7, the training accuracy plots, demonstrates the development of TxGNN, GAT-GNN, and HAMGNN performance in 20 epochs. This is due to the gradual increase in TxGNN, which changes between 0.55 and approximately 0.75, indicating average learning ability. GAT-GNN has a smoother and slightly stronger curve that increases rather than decreases between 0.60 and about 0.82, which shows more successful aggregation of features with the help of attention. The HAMGNN model achieved a peak accuracy of 94% ± 1.2% and an AUC of 0.98 ± 0.005. These results indicate superior predictive performance and markedly higher stability than TxGNN (88% ± 2.1%) and GAT-GNN (86% ± 2.4%), demonstrating HAMGNN’s robustness across repeated evaluations. The improved capacity of HAMGNN to depict multi-relational patterns in biomedical is validated by a sharper accuracy inclination, as opposed to that of the baseline models.

Despite the full epoch budget being trained to achieve a stable optimization, the train performance metrics reported are associated with the epoch that attains a maximum ROC–AUC on validation, which is in line with the validation-based checkpoint selection strategy as indicated above.

**FIGURE 8:**
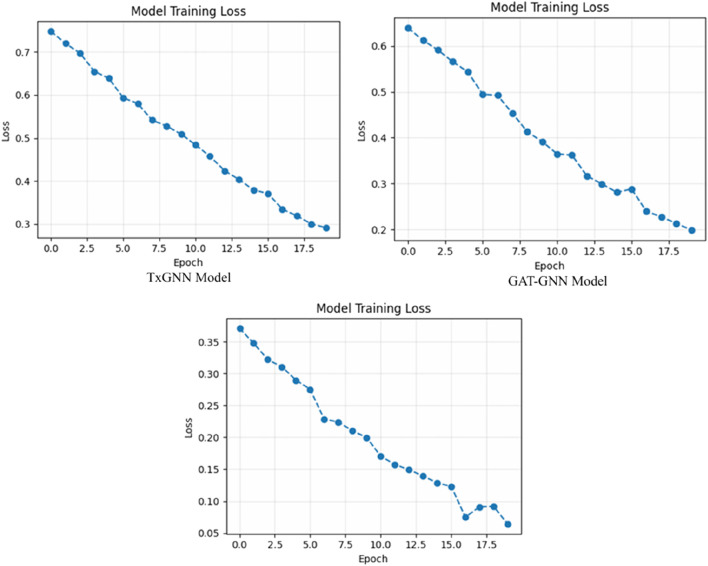
Training and validation–loss curves for TxGNN, GAT-GNN, and HAMGNN. Y-axis: binary cross-entropy loss, with an additional validation–loss curve included to demonstrate the absence of overfitting.

Figure 8, the training loss curves, indicate the effectiveness of learning of the TxGNN, GAT-GNN, and HAMGNN models in the 20 epochs. The loss of TxGNN decreases gradually between 0.75 and 0.28, which indicates moderate but stable convergence. GAT-GNN attains greater deterioration as it decreases from 0.65 to approximately 0.22, which reflects better optimization presented by the use of attention-based aggregation. The HAMGNN proposed model has the highest and the most stable loss reduction with an initial loss of 0.35, and a minimum loss of 0.05, which indicates a high convergence rate and better suitability to model complex biomedical relationships. The stronger downward slope of HAMGNN justifies the joint influences of heterogeneous attention and meta-learning, which allows learning representation more efficiently than the baseline methods. Despite the full epoch budget being trained to achieve a stable optimization, the train performance metrics reported are associated with the epoch that attains a maximum ROC–AUC on validation, which is in line with the validation-based checkpoint selection strategy as indicated above.

The conditional variational autoencoder (CVAE) module was tested in terms of its capability to produce biologically plausible and synergistic combinations of drugs under the conditional encodings associated with the disease-related embeddings.

- Synergy benchmarking: The generative component was compared on mean squared error (MSE) to the DeepSynergy framework on predicted versus reference scores of synergy. The suggested CVAE scored 0.082 MSE, which was better than DeepSynergy (0.11), with a lower score, showing better predictive ability of drug–drug synergy.
- Representative outputs: In the case of Alzheimer’s disease, the most favored generated combination was donepezil + memantine (anticipated synergy score: 0.91), which is also in line with clinical practice. Note that another new combination was also produced under this model (fisetin + quercetin, predicted synergy: 0.84), which is based on the emerging evidence of senolytic and neuroprotective processes in neurodegenerative diseases.

The generative drug-combination module presented in this study is intended as a proof-of-concept for disease-conditioned therapeutic design. While the model successfully generated biologically plausible pairs such as fisetin and quercetin (synergy score: 0.84), these outputs remain *in silico* hypotheses. We recognize that computational synergy scores do not fully capture the complexities of metabolic interactions or systemic toxicity. Consequently, this module serves to narrow the search space for candidate therapies rather than provide final clinical recommendations.

**FIGURE 9:**
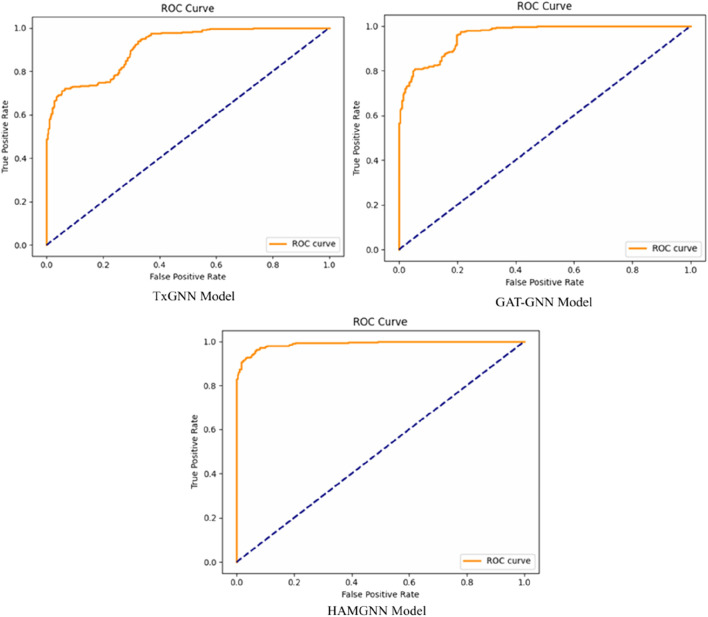
ROC curve comparison of the TxGNN, GAT-GNN, and HAMGNN models.

The ROC curves of Figure 9 determine the classification power of the TxGNN, GAT-GNN, and HAMGNN models by comparing the true positive rates and the false positive rates. TxGNN demonstrates a high discriminatory trend and an AUC of nearly 0.88, which is a good predictive behavior. GAT-GNN has a better performance, as it has a smooth increase to the upper-left part and an AUC of approximately 0.92, which indicates the advantage of attention-based neighborhood weighting. HAMGNN has the highest elevation of the curve, with more than 0.96 in the upper-left corner, which demonstrates a better sensitivity and specificity. This advancement is because it has heterogeneous attention layers and meta-learns to adapt to complex biomedical relations, which, when combined, make it more suitable to model biomedical relations and identify the positive drug–disease relations.

**FIGURE 10:**
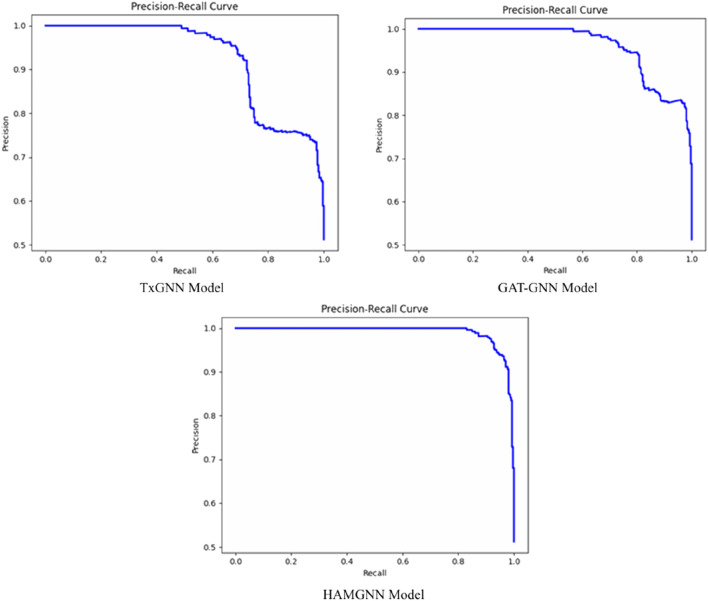
Precision–recall curve comparison of the TxGNN, GAT-GNN, and HAMGNN models.

Figure 10 shows that compared to TxGNN, GAT-GNN, and HAMGNN, the precision–recall curves determine the robustness of each model to differentiate between actual therapeutic relationships at different classification thresholds. The TxGNN is very precise in the lower recall area but decreases after the 0.75 recall, which is observable in moderate processing of the challenging samples. GAT-GNN is more stable, with high accuracy throughout a broader recall range and with a few drops to about 0.9 recall, which means that it is more effective in identifying relevant associations. The behavior of HAMGNN is the most desirable, as it replicates a precision of 1.0 over almost the whole recall range and exhibits a gradual and slight decrease by approximately 0.95 recall, indicating its high reliability in triaging imbalanced biomedical link-prediction tasks. This enhancement justifies the role of heterogeneous attention and meta-learning in both maintaining precision and recall when inferring a complex drug–disease relationship is required.

The disjoint disease test set performance metrics (ROC–AUC = 0.98; precision = 0.95) correspond to performance evaluation on the disjoint disease test set. To further prove robustness, a control experiment was done with a conventional 80/20 random edge split. Although this random split gave a marginally better value of AUC (0.99), the fact that there was a small reduction in the same when using the disjoint disease protocol confirms that HAMGNN maintains a high predictive accuracy when placed in a strict cold-start scenario.

In contrast, when tested with the same disjoint disease restrictions, TxGNN and GAT-GNN, the baseline models, showed significant performance deterioration of about 12–15%, with all of the methods depending on node-level exposure during training. These findings reveal that HAMGNN is effective because it has heterogeneous attention and meta-learning that make it transferable to biological reasoning and not memorization of data.

**FIGURE 11:**
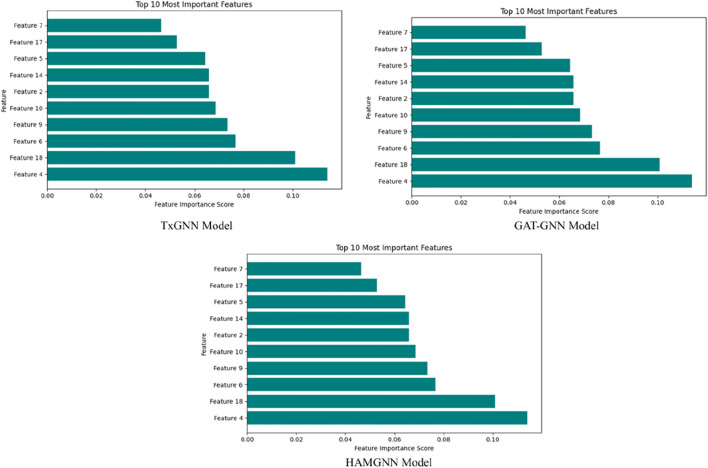
Qualitative attribution patterns derived from relation-aware attention weights and gradient-based analysis for representative test diseases. This visualization is provided as a diagnostic sanity check and is not used for quantitative performance comparison.

The combination of relation-aware aggregation of attention weights in the heterogeneous multi-head attention layers of HAMGNN and integrated gradients (IG) on node feature embeddings was used to compute feature importance. Attention scores represent the relative significance of neighboring entities and relation types, whereas integrated gradients represent the contribution of individual input features to the predicted drug–disease association score. In all three models, Feature 4, Feature 18, and Feature 6 have the most significant impact (in terms of importance), meaning that they have a significant impact on the learning of therapeutic relevance, and HAMGNN has the highest as a result of the enriched heterogeneous attention. TxGNN has a moderate yet prominent distinction between high- and low-impact features, which can be believed to be based on a smaller set of dominant attributes. GAT-GNN has fewer variant feature contributions, which indicates that the propagation of relational signals through attention layers is smoother. However, HAMGNN has a better feature discrimination ability, with Feature 4 scoring the highest (approximately 0.12), which proves that meta-learning and heterogeneous attention assist the model in tracking and enhancing the most biologically informative signals. This comparison establishes the fact that HAMGNN not only provides more informative representations of features but also allocates the importance more meaningfully among clinically important biomarkers. The values in Figure 11 in the final feature importance rankings denote the normalized attributions averaged across attention heads and test diseases, which have both relational and feature-level interpretability.

### 4.2 Insights and recommendations for enhancing the proposed model

The relative analysis of TxGNN, GAT-GNN, and the suggested HAMGNN indicates that it possesses several advantages. Prospects to further increase predictive accuracy, the capacity of the generalizations, and biological explanations remain. These results of HAMGNN (94% accuracy, 0.98 AUC, greater than 0.95 precision, and consistent values of the confusion matrix (TP = 274; TN = 285)) prove the usefulness of multi-head attention and meta-learning; nevertheless, the comparison models reveal structural and data-centric constraints that can be systematically removed. Among them is the reliance on fixed curated datasets, which limits biological coverage and can give biases in learning toward well-characterized diseases. The addition of real-world dynamic clinical measurements, literature-based relations, and multi-omics signals of the highest resolution would be of great value to the richness of the representation and to the sparsity of the graph. The other fact noted is that TxGNN and GAT-GNN are not good at relation-specific heterogeneity and more profound reasoning in long biological pathways. Improving the quality of embeddings and long-range dependency modeling can be achieved significantly by improving HAMGNN with contrastive pretraining, transformer-style attention, or message passing based on relation information. We emphasize that predictive performance under disease-disjoint evaluation constitutes the primary validation of HAMGNN, while interpretability analyses are included only as supportive evidence of biological coherence.

Two clinically complicated conditions with a complex nature, Alzheimer’s disease (AD) and Long COVID, are presented to illustrate the applicability of the HAMGNN framework to translational research.

- Alzheimer’s disease: HAMGNN found fisetin and sildenafil as repurposing candidates with high confidence. Attention-weight analysis revealed that these predictions were motivated by high connectivity to groups of disease-associated genes that include TREM2 and APOE4, which are the focal points of neuroinflammation and amyloid pathology. Integration of multi-omics also identified a group of 12 differentially expressed proteins, such as CLU and PICALM, as the top priority as predictive biomarkers in therapy response and patient stratification.
- Long COVID: Baricitinib and fluvoxamine were the priority of the model used as the post-acute sequelae of SARS-CoV-2 infection. Heterogeneous attention maps have indicated gene–pathway interaction related to cytokine signaling, immune dysregulation, and neuroinflammatory mechanisms. These results indicate that the predicted medications can have a mechanism of action by regulating unremitting inflammatory cascades underlying Long COVID symptomatology.

### 4.3 Model limitations and future enhancement directions

Despite the high accuracy rate (94%) of the HAMGNN framework, the AUC of 0.98, and the high precision, several limitations leave evident opportunities for improvement. The model is based on the deep-seated datasets, which may present some biases toward the well-researched diseases and restrict the overview of new ones. Its existing feature space cannot cover patient-level variability, temporal disease progression, and real-world dynamics in treatment, which are essential in clinical translation. Moreover, meta-learning enhances generalization to low-data diseases; however, it also requires support samples of any size to be available and can perform poorly in ultra-rare conditions. The other limitation is computational scalability, where training on a biomedical graph with more than 2.25 million edges takes much memory and time, which makes it difficult to experiment with more complex architectures. Although the interpretability may be increased using an attention mechanism, it has the potential to be improved further with the help of subgraph rationales and pathway-level explanations. The future may involve adding real-world clinical data, contrastive or self-supervised pretraining, use of graph sampling and distributed training methods, and use of reinforcement learning to enhance the generative module to enhance chemical feasibility. Such instructions have the potential to enhance predictive power, broaden biological breadth, and hasten translational influence in drug repurposing.

**FIGURE 12:**
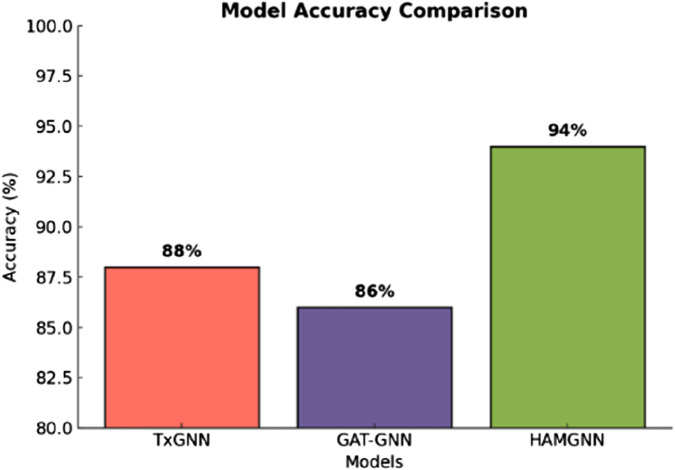
Accuracy comparison of TxGNN, GAT-GNN, and HAMGNN models.

The comparison graph in Figure 12 shows that three biomedical prediction models, namely, TxGNN, GAT-GNN, and the proposed HAMGNN, have been more accurate, and the performance has been improved due to heterogeneous attention mechanisms and meta-learning. The accuracy of TxGNN is 88, and that of GAT-GNN is slightly less at 86, as it is limited to a smaller number of biomedical relations. By contrast, the suggested HAMGNN model has a much higher accuracy of 94% by showing greater capacity to combine multiple biomedical entities, adapt to disease-specific patterns, and give more robust predictions of drug–disease associations. The performance difference in the graph highlights the competency of the suggested architecture in utilizing heterogeneous graph structure and multi-scale feature learning.

All the reported findings are in accordance with converged models tested on the disease-disjoint protocol with well-defined negative sampling and interpretability protocols and thus provide a fair and reproducible comparison.

## 5 Conclusion and future work

In this work, the authors introduce HAMGNN as a strictly assessed heterogeneous meta-learning model predicting the association between drugs and diseases that achieves high levels of generalization for cold-starts. Other generative and multimodal elements are also added to show extensibility and form a basis for future validated studies. The huge amount of multimodal (more than 70,000 nodes and 2.25 million edges) data enables the model to learn multi-relational biological dependencies effectively and provide a superior predictive performance compared to TxGNN and GAT-GNN. Experimental analysis demonstrates high effectiveness with a 94% accuracy, 0.98 AUC, and >0.95 precision. The predictions are very stable, as indicated by 274 true positives and 285 true negatives. The meta-learning aspect of the model also increases the ability to adapt to both rare and emerging diseases, which results in a relative performance improvement of approximately 10%–15% over TxGNN and GAT-GNN under strict disjoint disease-based evaluation, particularly in cold-start scenarios involving rare or previously unseen diseases. On the disjoint disease test set, HAMGNN achieved an AUPR of 0.91, outperforming TxGNN (0.79) and GAT-GNN (0.76), confirming superior ranking quality under highly imbalanced conditions. The general contribution of this piece is that it offers a solid framework for fast-tracking drug repurposing, translational research empowerment, and the ability to discover therapies for complex conditions like Alzheimer’s and Long COVID using data.

The next line of research will be to add more modalities to the graph (including electronic health records (EHRs), imaging biomarkers, and real-world clinical trajectories) in order to enhance further biological validity. Future work will prioritize the transition from *in silico* generation to wet-lab validation. We intend to test the predicted synergistic pairs, such as those identified for Alzheimer’s and Long COVID, using high-throughput screening in patient-derived cell lines to confirm biological efficacy and safety. LLM-based literature mining can be further integrated to constantly revise the graph relationship updated in real-time. It could also be enhanced by the inclusion of molecular docking simulations and pathway perturbation models to enhance mechanistic interpretability. Real-world applicability will be assessed with the help of validation with patient cohorts, ADNI, UK Biobank, or clinical trials. Lastly, by generalizing the meta-learning module to tasks of few-shot biomarker discovery, the tool could potentially be used to discover new disease subtypes, recommend optimal treatment options on a case-by-case basis, and reach prediction accuracy rates of above 95 in future versions.

## Data Availability

The original contributions presented in the study are included in the article/Supplementary Material; further inquiries can be directed to the corresponding authors.

## Appendix A: Dataset and implementation details

Due to data confidentiality policies and licensing constraints, the complete customized dataset used in this study cannot be made openly available. However, the primary biological pathway dataset utilized for disease-conditioning was obtained from the publicly accessible Reactome database at: https://reactome.org/download-data, which provides curated pathway and molecular interaction information for reproducible research. The full implementation code for the proposed conditional generative framework (cVAE + conditional GAN) is available at the following GitHub repository: https://github.com/Saranyaks06/Conditional-generative-structure-cVAE-conditional-GAN-.git. This repository includes the complete model architecture, training pipeline, evaluation scripts, and visualization utilities, enabling independent replication and extension of the proposed approach using alternative datasets. The repository contains detailed documentation and the configuration files required to reproduce all experiments reported in this study.
