# Supplementary figures and images for: Generative AI in drug repurposing and biomarker discovery: a multimodal approach
Source: Front Bioinform. 2026 Mar 20;6:1755412. doi: 10.3389/fbinf.2026.1755412 (PMC13047114; doi:10.3389/fbinf.2026.1755412)

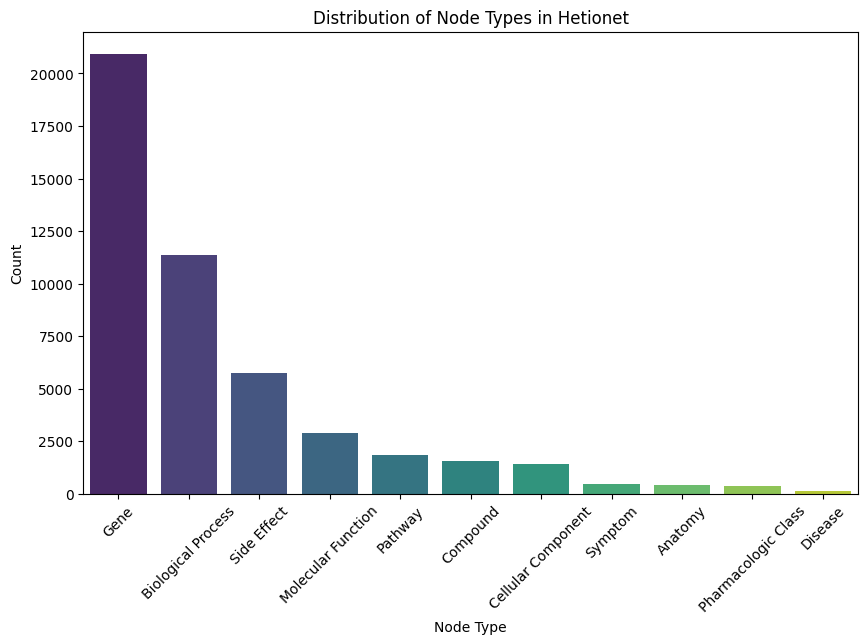

Supplement: Supplementary file 1 [file DataSheet1.zip › images_300dpi/image_1.png]

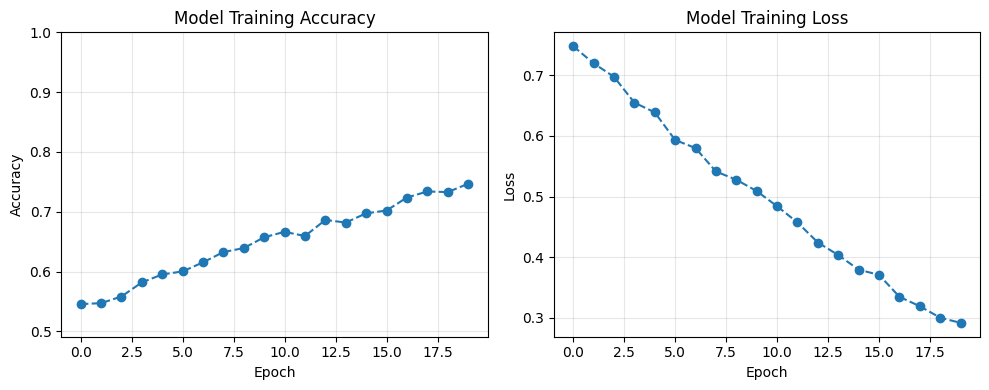

Supplement: Supplementary file 1 [file DataSheet1.zip › images_300dpi/image_10.png]

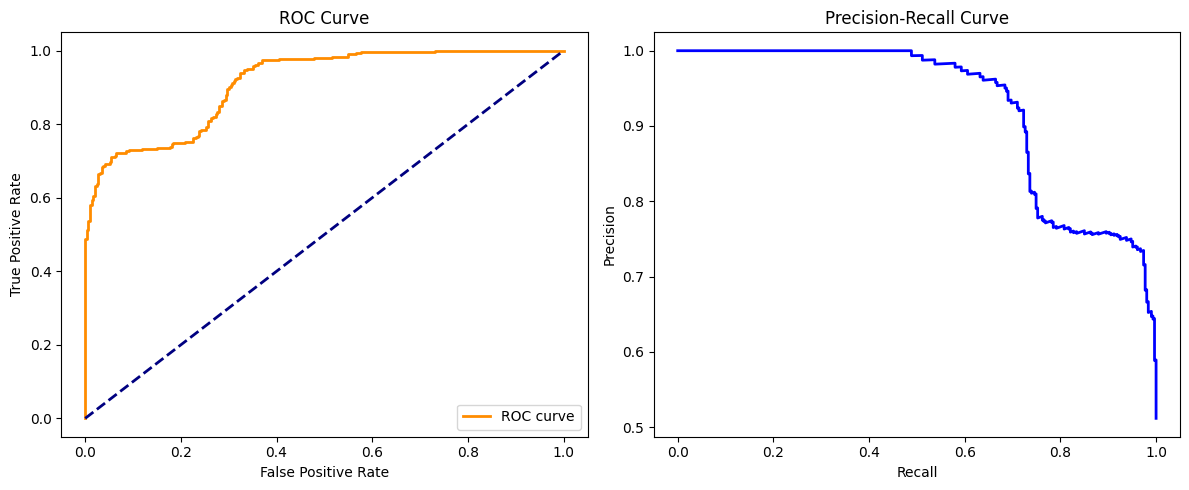

Supplement: Supplementary file 1 [file DataSheet1.zip › images_300dpi/image_11.png]

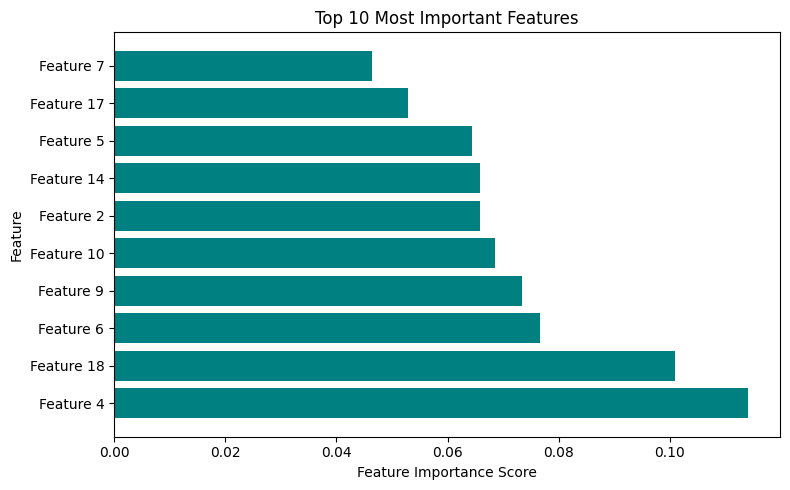

Supplement: Supplementary file 1 [file DataSheet1.zip › images_300dpi/image_12.png]

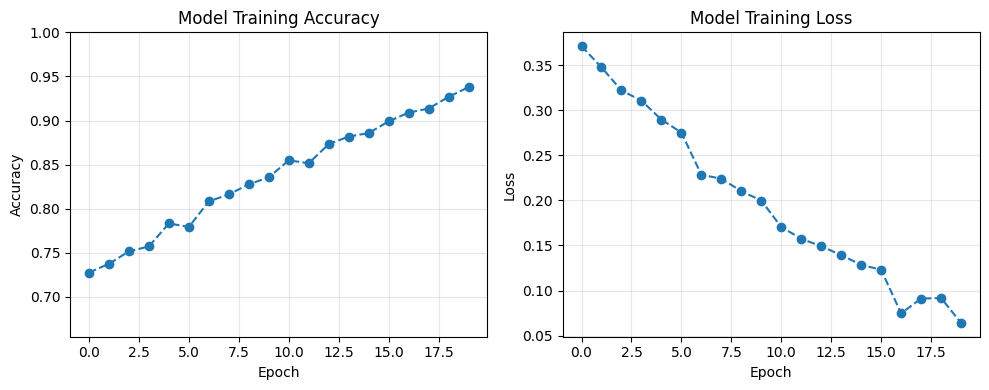

Supplement: Supplementary file 1 [file DataSheet1.zip › images_300dpi/image_13.png]

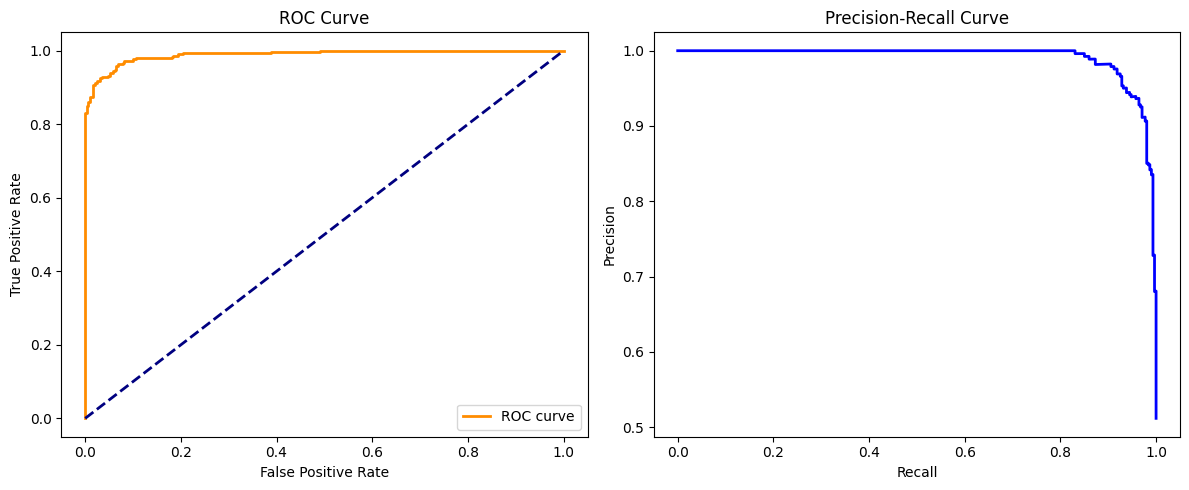

Supplement: Supplementary file 1 [file DataSheet1.zip › images_300dpi/image_14.png]

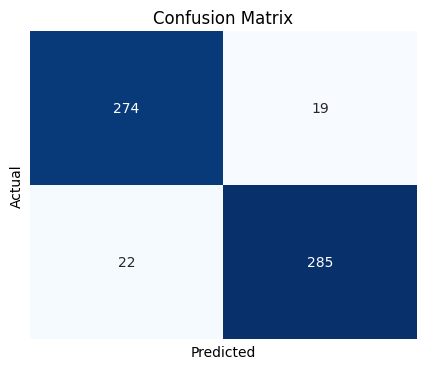

Supplement: Supplementary file 1 [file DataSheet1.zip › images_300dpi/image_15.png]

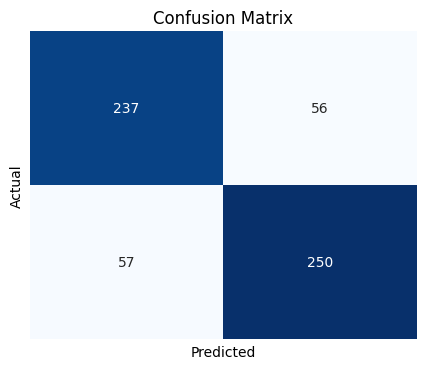

Supplement: Supplementary file 1 [file DataSheet1.zip › images_300dpi/image_16.png]

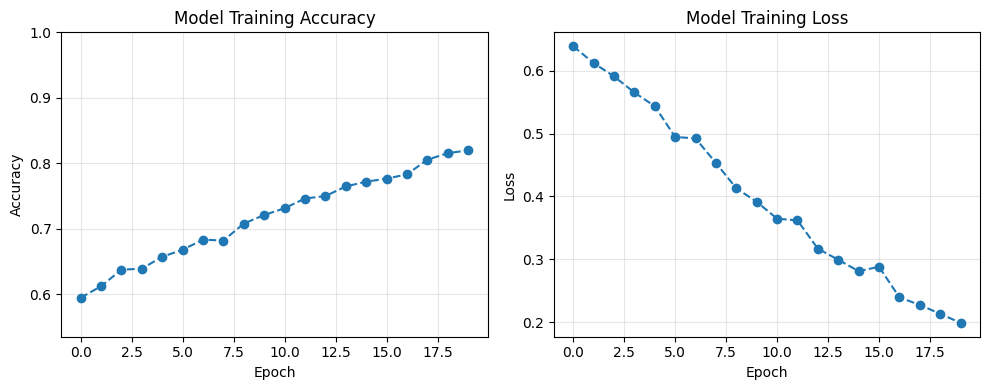

Supplement: Supplementary file 1 [file DataSheet1.zip › images_300dpi/image_17.png]

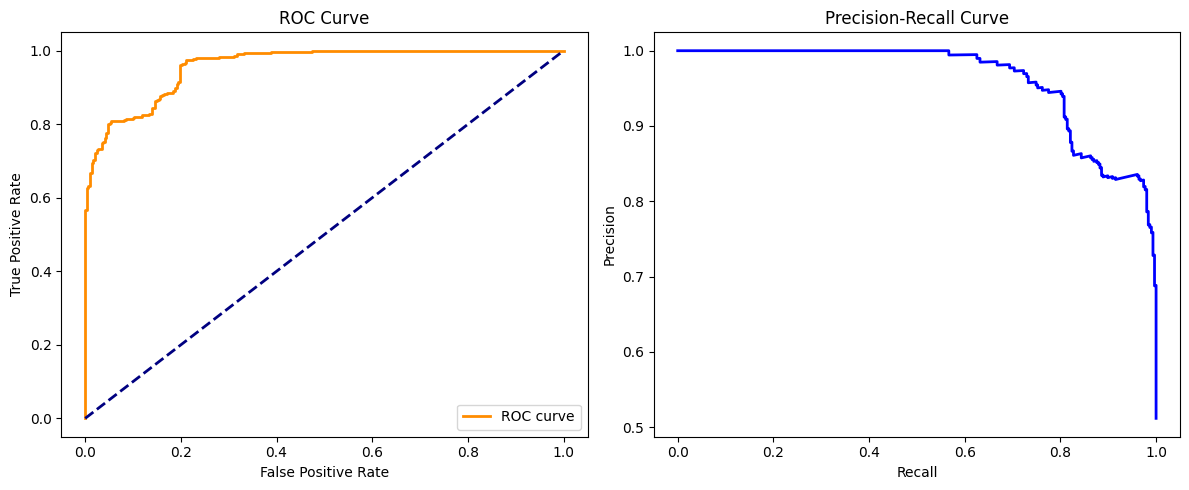

Supplement: Supplementary file 1 [file DataSheet1.zip › images_300dpi/image_18.png]

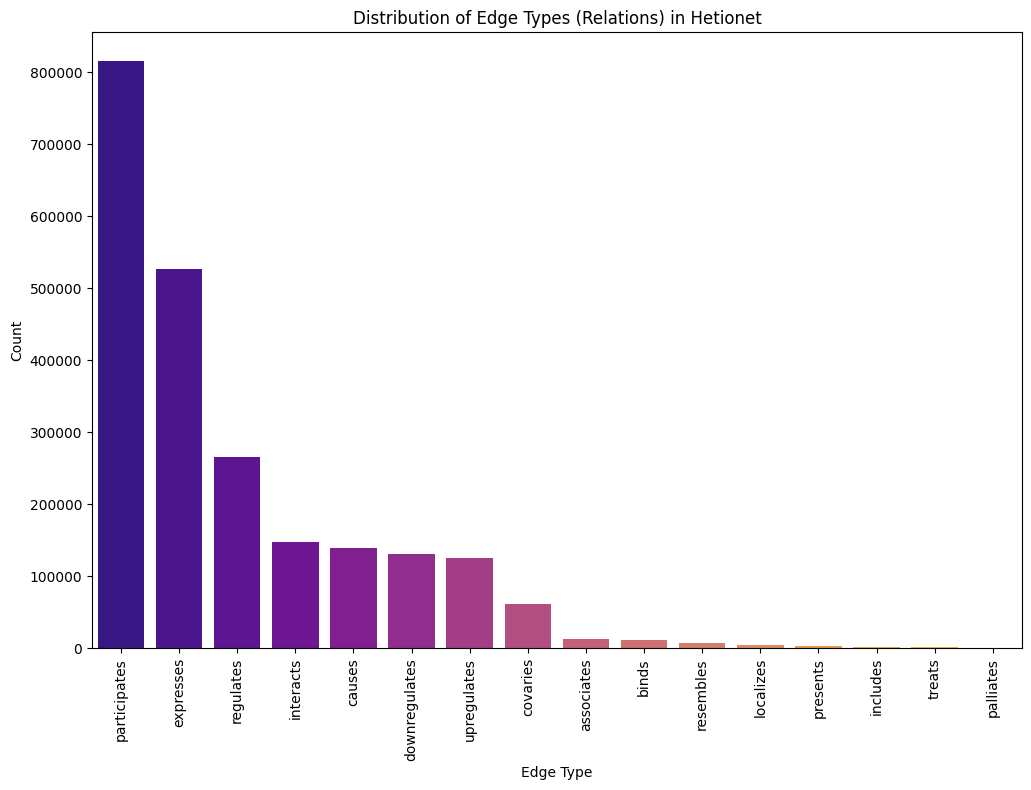

Supplement: Supplementary file 1 [file DataSheet1.zip › images_300dpi/image_2.png]

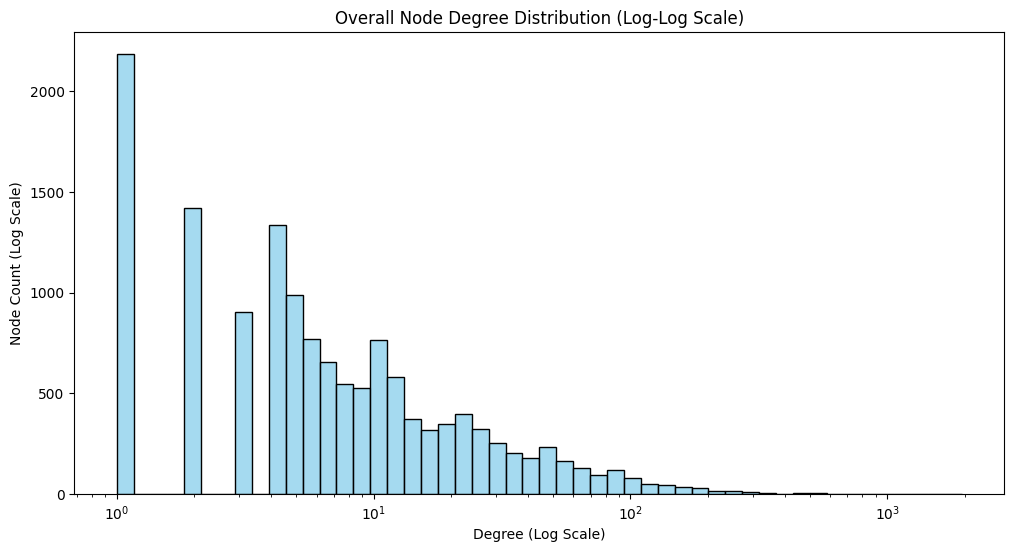

Supplement: Supplementary file 1 [file DataSheet1.zip › images_300dpi/image_3.png]

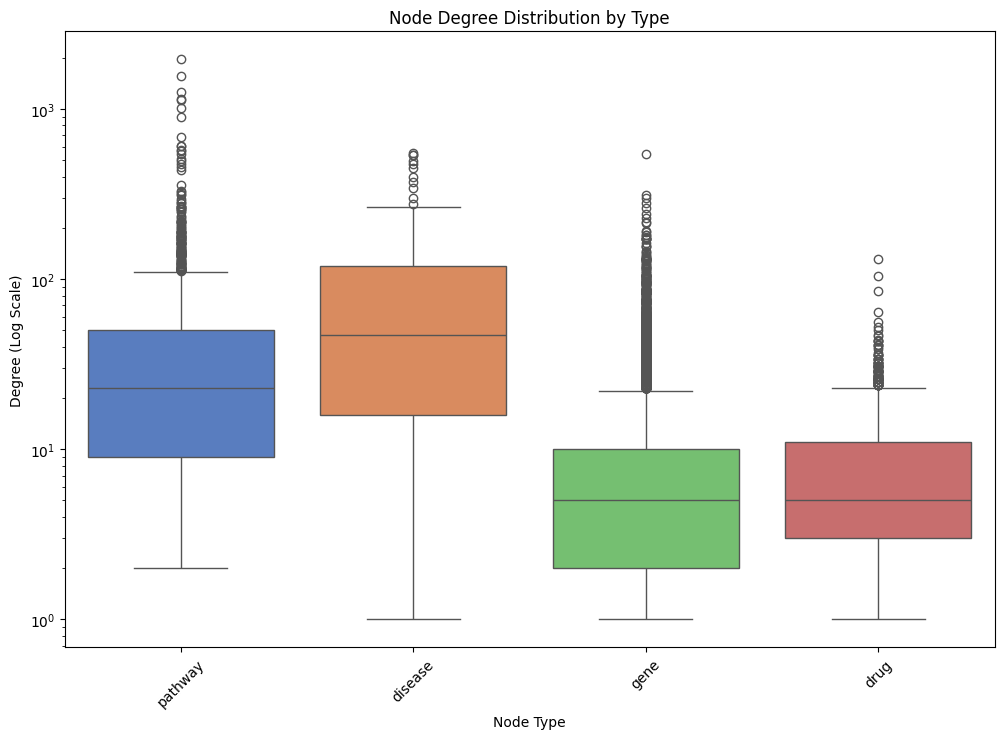

Supplement: Supplementary file 1 [file DataSheet1.zip › images_300dpi/image_4.png]

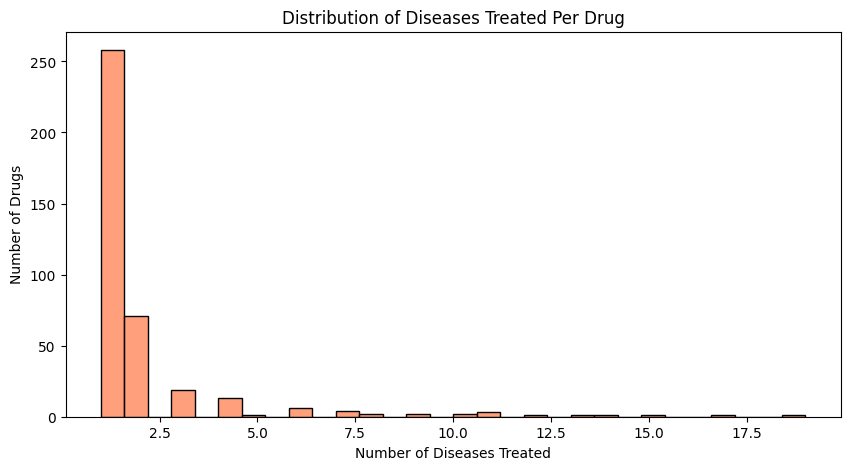

Supplement: Supplementary file 1 [file DataSheet1.zip › images_300dpi/image_5.png]

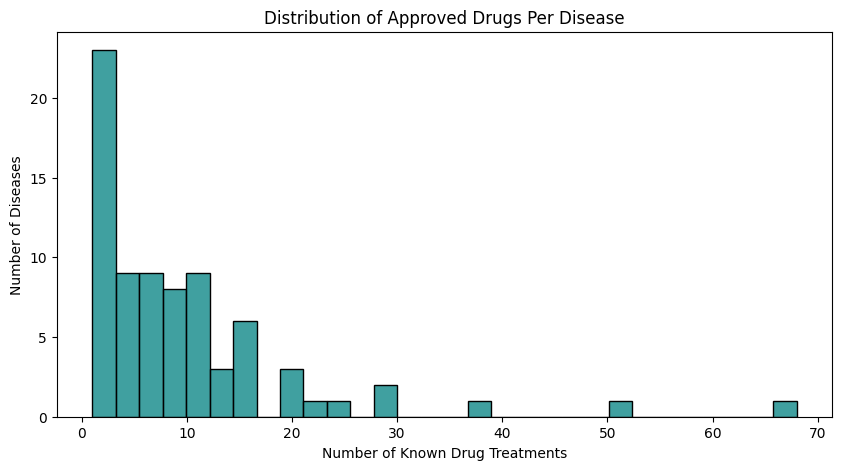

Supplement: Supplementary file 1 [file DataSheet1.zip › images_300dpi/image_6.png]

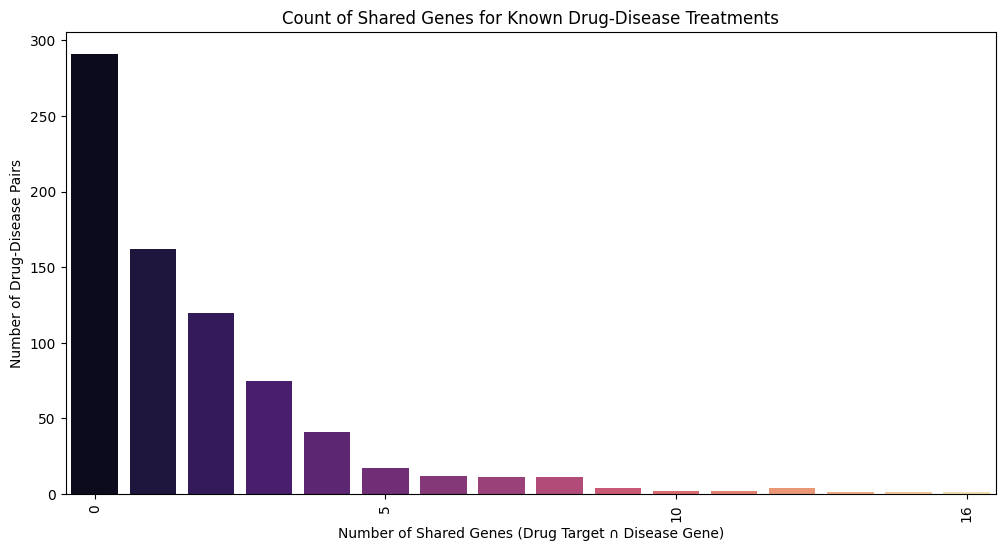

Supplement: Supplementary file 1 [file DataSheet1.zip › images_300dpi/image_7.png]

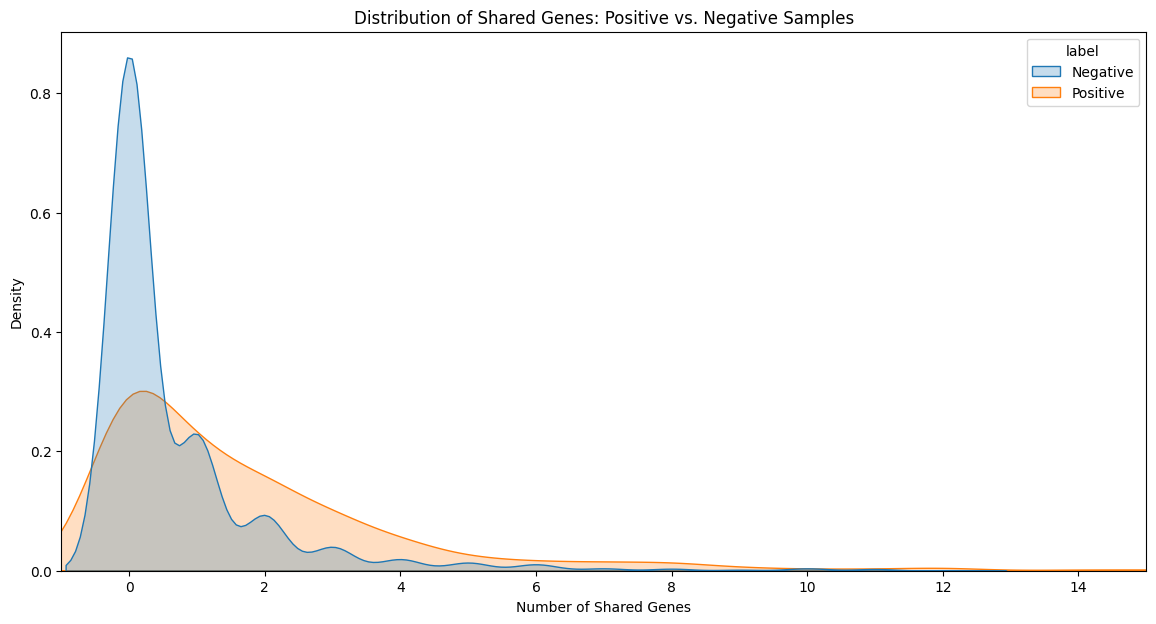

Supplement: Supplementary file 1 [file DataSheet1.zip › images_300dpi/image_8.png]

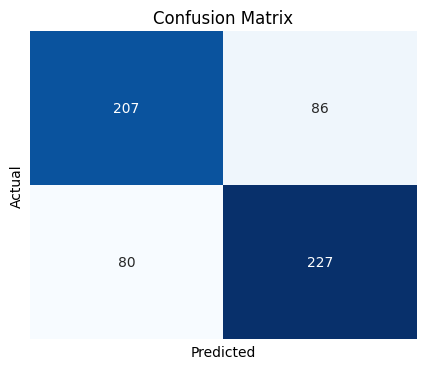

Supplement: Supplementary file 1 [file DataSheet1.zip › images_300dpi/image_9.png]
